# Genome-wide identification of germin-like proteins in peanut (*Arachis hypogea* L.) and expression analysis under different abiotic stresses

**DOI:** 10.3389/fpls.2022.1044144

**Published:** 2023-01-23

**Authors:** Qiang Yang, Yasir Sharif, Yuhui Zhuang, Hua Chen, Chong Zhang, Huiwen Fu, Shanshan Wang, Tiecheng Cai, Kun Chen, Ali Raza, Lihui Wang, Weijian Zhuang

**Affiliations:** ^1^ Center of Legume Plant Genetics and System Biology, College of Agronomy, College of Life Science, Fujian Agriculture and Forestry University (FAFU), Fuzhou, Fujian, China; ^2^ College of Plant Protection, Fujian Agriculture and Forestry University (FAFU), Fuzhou, China

**Keywords:** phylogenetic relations, gene evolution, environmental stress, functional annotation, micro-RNAs

## Abstract

Peanut is an important food and feed crop, providing oil and protein nutrients. Germins and germin-like proteins (GLPs) are ubiquitously present in plants playing numerous roles in defense, growth and development, and different signaling pathways. However, the GLP members have not been comprehensively studied in peanut at the genome-wide scale. We carried out a genome-wide identification of the *GLP* genes in peanut genome. *GLP* members were identified comprehensively, and gene structure, genomic positions, motifs/domains distribution patterns, and phylogenetic history were studied in detail. Promoter Cis-elements, gene duplication, collinearity, miRNAs, protein-protein interactions, and expression were determined. A total of 84 *GLPs* (*AhGLPs *) were found in the genome of cultivated peanut. These *GLP* genes were clustered into six groups. Segmental duplication events played a key role in the evolution of *AhGLPs*, and purifying selection pressure was underlying the duplication process. Most *AhGLPs* possessed a well-maintained gene structure and motif organization within the same group. The promoter regions of *AhGLPs* contained several key cis-elements responsive to ‘phytohormones’, ‘growth and development’, defense, and ‘light induction’. Seven microRNAs (miRNAs) from six families were found targeting 25 *AhGLPs*. Gene Ontology (GO) enrichment analysis showed that *AhGLPs* are highly enriched in nutrient reservoir activity, aleurone grain, external encapsulating structure, multicellular organismal reproductive process, and response to acid chemicals, indicating their important biological roles. *AhGLP14, AhGLP38, AhGLP54,* and *AhGLP76* were expressed in most tissues, while *AhGLP26, AhGLP29,* and *AhGLP62* showed abundant expression in the pericarp. *AhGLP7, AhGLP20,* and *AhGLP21,* etc., showed specifically high expression in embryo, while *AhGLP12, AhGLP18, AhGLP40, AhGLP78*, and *AhGLP82* were highly expressed under different hormones, water, and temperature stress. The qRT-PCR results were in accordance with the transcriptome expression data. In short, these findings provided a foundation for future functional investigations on the *AhGLPs* for peanut breeding programs.

## 1 Introduction

Germin-like proteins (GLPs) are a group of pervasive water-soluble glycoproteins found in monocots, dicots, and gymnosperms (Bernier and Berna, 2001). *GLP* genes are widely distributed in the plant kingdom, and most of the genes are present in the form of multiple copies in plant genomes (Manosalva et al., 2009). Germins and germin-like proteins (*GLPs*) were initially discovered as germination-specific markers in germinating wheat seedlings (Lane et al., 1993). *GLPs* belong to the “Cupin superfamily” possessing β-sheet barrel (jellyroll beta-barrel structural domain) cupin domain (PF00190) with metal ion binding site at their C-terminus (Agarwal et al., 2009), and generally, these genes are encoded by two exons. Cupin superfamily is a functionally diverse family (Dunwell et al., 2004) with seed storage-related functions, such as vicilins (Gane et al., 1998). Members of this protein family contain two conserved motifs known as “germin box” (Lane et al., 1991; Yamahara et al., 1999). Both motifs of Germin-like proteins “G(x)5HxH(x)3,4E(x)6G” and “G(x)5PxG(x)2H(x)3N” are packed in a classic jellyroll beta-barrel structural domain (Woo et al., 2000). Crystallographic studies of barley germin proteins elaborated that six germin proteins form a stable hexamer structure, with each protein binding to a manganese ion (Woo et al., 2000). The monomer subunits of hexamer structure form “trimers of dimers,” in which ligands bound with the manganese ions similarly to that of manganese superoxide dismutase (Carter and Thornburg, 2000). Each monomer also contains an irregular extension at the N-terminus, and the domain shape of the N-terminus is generally conserved in many GLPs. Hexamer structure is composed of almost 1200 amino acid residues with a molecular weight of approximately 13 kDa (Lane, 2002), but on the contrary, a single copy of *GLP* has been discovered in rice which is capable of SOD activity even in its dimeric form (Banerjee and Maiti, 2010). Classification of germins and GLPs is challenging due to their structural and sequence similarities (Agarwal et al., 2009). Generally, the “true germins” are a group of well-conserved homogenous proteins uniquely found in cereals (Bernier and Berna, 2001; Davidson et al., 2009), while the germin-like proteins are heterogeneous proteins with wider distribution in the plant kingdom (Woo et al., 2000; Dunwell et al., 2008).

Germins and *GLPs* play a wide range of functions in plants, and mostly, their functions are associated with enzymatic reactions, stress responses, and cell wall synthesis (Dunwell et al., 2000; Sun et al., 2020). Members of this gene family are highly expressed under various biotic stresses, including bacterial pathogens responses such as bacterial rust resistance in peanut (Wang et al., 2013), fungal pathogens including powdery mildew (*Erysiphe necator* infection) responses in grapevine (Godfrey et al., 2007), *Aspergillus flavus* response in peanut (Knecht et al., 2010; Rietz et al., 2012), and viral pathogens responses in tobacco (Guevara-Olvera et al., 2012). *GLPs* have been involved in defense responses against fungal pathogens in cereals (Zimmermann et al., 2006). Transgenic tobacco and *Arabidopsis* plants overexpressing soybean and sunflower *GLPs* showed improved resistance to *Sclerotinia sclerotiorum* (Beracochea et al., 2015; Zhang et al., 2018). Transient overexpression of barley and wheat *GLP4* (*HvGLP4* and *TaGLP4*) increased resistance to *B. graminis* in transgenic *Arabidopsis* plants (Christensen et al., 2004). A newly discovered *GLP* member from upland cotton (*Gossypium hirsutum*) *GhABP19* showed increased resistance against fungal pathogens, *Fusarium oxysporum* and *Verticillium dahliae* in transgenic *Arabidopsis* plants (Pei et al., 2019). *GLPs* are also highly expressed under abiotic stresses, including drought stress, salt stress, wound stress (Wang et al., 2013), high-temperature stress (Gangadhar et al., 2021), and heavy metals stress (Houde and Diallo, 2008). Recent studies have shown that *OsGLP1* from rice helps plant acclimatize to UV radiations (He et al., 2021). *GLPs* also have regulatory effects in certain growth or development-related pathways, as it has been reported that a member of the *GLP* family in *Gossypium barbadense* (*GbGLP2*) is involved in secondary cell wall growth and ultimately controls fiber length (Sun et al., 2020).

Although some *GLPs* from various crop species have been studied in detail, the functions of most *GLPs* are unknown and uncharacterized. Mostly plant *GLPs* with unknown functions have been classified into various subfamilies (Carter and Thornburg, 1999; Carter and Thornburg, 2000). The true germins subfamily (cereals) contains the proteins with oxalate oxidase activity, while the members of subfamilies 1 and 2 are attributed with superoxide dismutase activity (SOD). Subfamily 3 includes the proteins with phosphodiesterase activity (3.1.4.1), while some studies have reported more subdivisions (Nakata et al., 2004). As in the case of barley, five subfamilies (*HvGER1*-*HvGER5*) have been reported (Lu et al., 2010).

Keeping in view the above reports, it is a fact that germins and *GLPs* are an important group of plant proteins playing a broad-spectrum role in plant growth regulation and defense responses. Despite the fact that *GLPs* have been comprehensively studied in some important plant species, including soybean (Lu et al., 2010), *Arabidopsis* (Rietz et al., 2012), rice (Li et al., 2016), and wheat (Yuan et al., 2021) and some of their members have been functionally characterized. Still, this gene family was yet to be comprehensively described in the peanut genome. Peanut (*Arachis hypogea* L.) is a popular food crop, and more than one hundred countries share its cultivation. It is an important source of vegetable oil, proteins, minerals, and dietary fibers (Toomer, 2018). Several biotic and abiotic stress agents affect the peanut plant throughout its lifecycle (Ali et al., 2020). Identification and application of novel genetic, genomics and gene editing resources can help to improve peanut growth and stress resistance (Yaqoob et al., 2023).

Peanut is now a resource-rich legume crop with massive transcriptome data (Clevenger et al., 2016; Sinha et al., 2020; Gangurde et al., 2021). Trait dissection and candidate gene discovery with linkage and association mapping attempted for several traits, for instance, yield traits (Gangurde et al., 2020; Pandey et al., 2020; Jadhav et al., 2021), fresh seed dormancy (Gangurde et al., 2020; Kumar et al., 2020; Bomireddy et al., 2022), disease resistance (Dodia et al., 2019; Shasidhar et al., 2020), and aflatoxin contamination (Pandey et al., 2019; Khan et al., 2020; Soni et al., 2020). The utilization of the available resources with new data is essential. In the present study, we used publicly available transcriptome datasets to investigate the expression levels of *GLPs*. The members of the *GLP* family in the genomes of cultivated peanut and its wild parents (*Arachis duranensis* and *Arachis ipaensis*) were identified by available genome annotations. The genomic distribution, conserved motifs organization, *cis*-regulatory elements, phylogenetic relationships, protein physicochemical properties, functional annotation (GO enrichment), prediction of miRNAs targeting the *AhGLPs*, and expression patterns in different tissues and under stress conditions were studied in detail. Expression patterns of selective genes were studied under different abiotic stresses by qRT-PCR analysis. This study will help to understand the role of *GLPs* in peanut against various biotic and abiotic stresses, growth and development and provide basic information for future studies.

## 2 Materials and methods

### 2.1 Identification of germin-like proteins in peanut

Germin-like proteins were identified in a systematic way in peanut. First of all, *Arabidopsis GLPs* (*Arabidopsis thaliana* TAIR10-Thales cress) protein sequences were obtained from the Phytozome server (https://phytozome-next.jgi.doe.gov/) (Goodstein et al., 2012). The *GLPs* in diploid progenitors (*A. duranensis* and *A. ipaensis*) were searched from the PeanutBase database (https://www.peanutbase.org/home) (Bertioli et al., 2016) by BLASTp search by using *AtGLPs* protein sequences as queries. GLPs in diploid progenitors were also searched from the Legume Information System (https://legumeinfo.org/), using the keyword search “germin” and “PF00190” (Pfam accession number of Germin-like proteins). The protein sequences of identified GLPs from *A. duranensis*, *A. ipaensis*, and *A. thaliana* were used to search the *GLPs* in cultivated peanut from the Peanut Genome Resource (PGR) database (http://peanutgr.fafu.edu.cn/) (Zhuang et al., 2019). Cultivated peanut *GLPs* (*AhGLPs*) were also searched by keyword search “germin” in the PGR database. Finally, all identified proteins were checked for the presence of germin domain by Batch CD-search at NCBI (https://www.ncbi.nlm.nih.gov/). Duplicated IDs and different splicing variants of the same gene were removed to get the unique IDs.

### 2.2 Chromosomal location and phylogenetic analysis

The information about the chromosomal distribution of *AhGLPs* was obtained from the PGR database (http://peanutgr.fafu.edu.cn/). TBtools software (Chen et al., 2020) was used to map out the genes on chromosomes. The evolutionary relationships of *AhGLPs* with their homologs in *A. duranensis* (*AdGLPs*), *A. ipaensis* (*AiGLPs*), and model dicot plant *A. thaliana* (*AtGLPs*) were assessed by evolutionary phylogenetic analysis. Multiple sequence alignment analysis was performed by the ClustalW algorithm (Thompson et al., 2003), and a Neighbor-Joining phylogenetic tree (Saitou et al., 1987) with 1000 bootstrap replications was constructed in MEGA-X software (Kumar et al., 2018) with the Poisson model. The phylogenetic tree was beautified *via* the online program iTOL v6 (Letunic and Bork, 2021), available at (https://itol.embl.de/).

### 2.3 Analysis of conserved motifs and gene structure

The gene structure (exon-intron distribution) analysis for *AhGLPs* was performed with the help of TBtools software by deploying the General Feature File (GFF3) downloaded from the PGR database (Zhuang et al., 2019). Conserved motifs of GLP genes were identified by MEME Suite (https://meme-suite.org/meme/) (Bailey et al., 2015) by employing the protein sequences. Parameters for motifs identification were set as; 6-200 optimum width range, the maximum number of Motifs: 10.

### 2.4 Prediction of physicochemical properties

Subcellular localization of *AhGLPs* in different cell compartments was predicted by CELLO v2.5 (http://cello.life.nctu.edu.tw/) (Yu et al., 2006), and other physiochemical properties, including molecular weight (MW), theoretical Isoelectric point (PI), were predicted by Expasy server (https://web.expasy.org/protparam/) (Gasteiger et al., 2003; Gasteiger et al., 2005).

### 2.5 *Cis-*regulatory elements analysis

For the analysis of *cis-*regulatory elements of *AhGLPs*, the promoter sequences (2kb upstream of the start codon) were scanned at the PlantCARE database (http://bioinformatics.psb.ugent.be/webtools/plantcare/html/) (Lescot et al., 2002). Promoter sequences were accessed from the PGR database. *Cis*-elements were divided into four main categories based on their functions. These categories include light-responsive elements, hormones-responsive elements, growth and development-related elements, and stress-responsive elements.

### 2.6 Synteny analysis, gene duplication, and orthologous gene clusters identification

The evolutionary genome conservations between three peanut species and *Arabidopsis* were analyzed by performing a comparative synteny analysis. The genome and GFF3 files of all these species were scanned for McScanX at TBtools software, and the resulting files were used for multiple synteny plots. To study the gene duplication in cultivated peanut, duplicated genes were identified by their phylogenetic relations and running an MCScanX for the whole peanut genome. The synonymous (Ks) and non-synonymous (Ka) substitution rates (Ka: No. of non-synonymous substitutions per non-synonymous site, Ks: No. of synonymous substitutions per synonymous site) were calculated by simple Ka/Ks calculator at TBtools software. Divergence time for duplicated gene pairs was calculated as ‘t=Ks/2r’ with the neutral substitution rate of r=8.12×10^-9^ (Bertioli et al., 2016). The orthologous GLP proteins in *A. hypogea*, *A. duranensis*, *A. ipaensis*, and *A. thaliana* were identified through orthovenn2 (https://orthovenn2.bioinfotoolkits.net/home) (Xu et al., 2019). Protein sequences of *Arabidopsis* and three peanut species were used to identify orthologous genes. The peanut species were assessed individually with each other and with *Arabidopsis* to identify orthologous gene clusters.

### 2.7 Prediction of miRNAs targeting peanut *GLPs*

The coding sequences of *AhGLPs* were used to predict the putative miRNAs targeting the *AhGLPs* through the psRNATarget database (https://www.zhaolab.org/psRNATarget/) (Dai et al., 2018) following the default settings. The schematic diagram showing the interaction networks between miRNAs and *AhGLPs* was drawn by Cytoscape software version 3.8.2 (https://cytoscape.org/) (Shannon et al., 2003).

### 2.8 Functional annotation and prediction of protein-protein interactions

We performed the gene ontology (GO) analysis of *AhGLPs* to predict their functional annotation. For that purpose, *AhGLP* proteins were scanned at the EggNOG database (http://eggnog-mapper.embl.de/). GO enrichment analysis was performed in TBtools software from predicted GO annotations.

Protein-protein interactions were predicted based on studied *Arabidopsis GLPs*. STRING 11.5 tool (https://www.string-db.org/cgi/) was used to construct the interaction network between peanut and *Arabidopsis* GLPs. The top 10 interactions were predicted with a medium threshold level (0.4). MCL clustering with inflation parameter 10 was used, and dotted lines were used between cluster edges.

### 2.9 Expression analysis of *AhGLPs*

Transcriptome expression data were used to view the expression patterns of *AhGLPs* in different tissues, under phytohormones, water, and temperature treatments. Transcriptome expression data for different tissues (leaf, stem, stem tip, Inflorescence, root, root and stem, root tip, root nodule, gynophore/peg, pericarp, testa, cotyledons, and embryo), hormones (ABA, SA, Brassinolide, paclobutrazol, ethephon, and ddH_2_O), water (drought and normal irrigation) and temperature treatments (low temperature and room temperature) were accessed from the Peanut Genome Resource database (Zhuang et al., 2019). The tissue samples were taken at different growth stages, and their RNA was mixed for transcriptome analysis. For hormone treatment, samples were collected at different time points (3h, 6h, 12h, 24h, and 48h). For drought stress, peanut plants at the flowering stage (eight leaves) were exposed to stress by withholding the water, and samples were taken after 3d, 6d, 9d, and 12d after drought treatments. For temperature stress, young plants (four leaves) were kept at 4°C, and samples were taken at 3h, 6h, 12h, 24h, and 48h. RNA samples of different time points were mixed for transcriptome analysis. The log2 normalization Fragments Per Kilobase Million (FPKM) of *AhGLPs* were used for expression analysis, and log2 normalization values were used to construct the expression heatmaps.

### 2.10 Stress treatments and qRT-PCR analysis

For stress treatments, a widely cultivated peanut variety, Minhua-6 “M-6” seeds were grown in small plastic pots. At four-leaf stage, seedlings were treated with ABA (10 µg/mL) and low temperature (4°C). Samples were collected at 3, 6, 9, and 12 hours after stress treatments, while non-treated leaf samples (0 h) were taken as control (CK). RNA was extracted with the CTAB method with some modifications (Sharif et al., 2022) and cDNA was synthesized with the help of Evo M-MLV RT Kit (Hunan Aikerui Biological Engineering Co., Ltd. China) according to manufacturer guidelines. Peanut *Actin* gene was used as internal control, while qRT-PCR was performed as per our previous study (Sharif et al., 2022). Relative expression levels of selected genes were calculated by 2^-ΔΔCT^ method (Livak and Schmittgen, 2001). Primers used for qRT-PCR are given in **Supplementary Table 9**. The graphs were drawn with GraphPad Prism 7.0 (Swift, 1997). The qRT-PCR results for the expression of selected genes under ABA and low temperature at different time points were subjected to analysis of variance (ANOVA) and Tuckey’s HSD test, to find the significant expression differences.

## 3 Results

### 3.1 Genome-wide identification and localizations of peanut *GLPs*

Thirty-seven *GPLs* in *A. duranensis* (*AdGLPs*) (V14167) (**Supplementary Table 1**) and 32 *GLPs* in *A. ipaensis* (*AiGLPs*) (K30076) (**Supplementary Table 2**) were identified as containing the germin domain PF00190. Then, 84 *GLPs* were found in *A. hypogaea* (*AhGLPs*) with the help of *AtGLPs*, *AdGLPs*, and *AiGLPs*. Previously, no systematic way to rename GLPs was available, and GLPs are mostly renamed according to their chromosomal/genomic locations. We renamed genes based on the genomic positions and chromosomal locations. Renaming based on genomic position as *AhGLP1*-*AhGLP84* was further used for analysis. Similarly, 37 *GLPs* of *A. duranensis* were renamed as *AdGLP1*-*AdGLP37*, and 32 GLPs of *A. ipaensis* were renamed as *AiGLP1*-*AiGLP32*.

**Figure 1:**
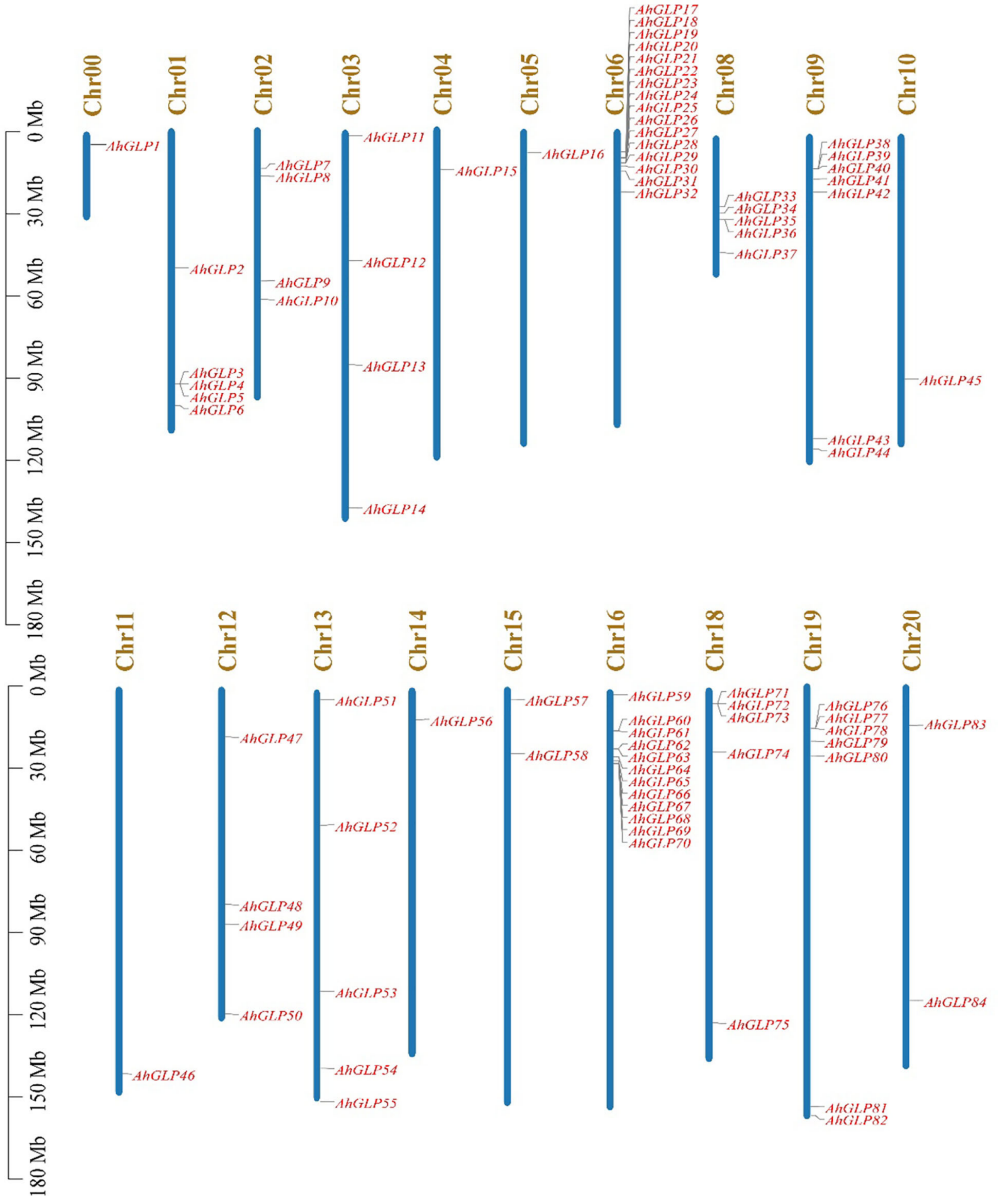
Chromosomal distribution of *Arachis hypogaea* Germin-like protein (*AhGLPs*) genes. The scale on left side indicates the chromosome length.

Germin-like proteins were unevenly distributed in the genome of cultivated peanut *A. hypogea*. The largest number of *AhGLPs* were present on chromosome Chr06 (16 out of 84), followed by 12 *GLPs* on the Chr16; 7 *GLPs* on Chr9 and Chr19 each; 5 *GLPs* on Chr01, Chr08, Chr13, and Chr18 each; 4 *GLPs* on Chr02, Chr03, Chr12 each; 2 *GLPs* on Chr15, Chr20 each. Chr04, Chr05, Chr10, Chr11, Chr14 possessed one *GLP* each. The chromosomal distribution patterns of *AhGLPs* are shown in **Figure 1**. One of the *AhGLPs* was present in the unassembled genome region. The genomic length of *AhGLPs* varied from 522 bp for *AhGLP10* to 99291 bp for *AhGLP29*. The CDS length, protein length, and molecular weight of *AhGLPs* ranged from 522-2145 bp, 173-714 amino acids, and 18.74 to 81.40 KDa, respectively. Their theoretical isoelectric points varied from 5.01 (*AhGLP73*) to 9.42 (*AhGLP29*). Subcellular localization prediction of *AhGLPs* showed their distribution in different cell compartments, including the plasma membrane, nucleus, cytoplasm, chloroplast, mitochondria, and extracellular spaces. A large number of proteins are localized in more than one cell organelles. Detailed information on the physicochemical properties of *AhGLPs* is given in **Table 1**. The genomic distribution of *GLPs* in the diploid peanut species (*A. duranensis* and *A. ipaensis*) is shown in **Supplementary Figures 1, 2**. The genomic, CDS, protein lengths, and other physiochemical properties of *GLPs* in *A. duranensis* and *A. ipaensis* are given in **Supplementary Tables 1, 2**. Protein sequences of *AhGLPs*, *AdGLPs*, *AiGLPs*, and *AtGLPs* are given in **Supplementary File 1**.

**Table 1:** Germin-like protein members in *Arachis hypogaea* and their physicochemical properties.

mRNA ID	Renamed^*^	Gene^**^	Chr	Gene (bp)	CDS (bp)	Exons	Protein aa	MW (KDa)	pI	Subcellular localization
AH00G03400.1	*AhGLP0-1*	*AhGLP1*	0	2188	1440	5	479	53.83764	5.61	Cytoplasmic
AH01G16460.1	*AhGLP1-1*	*AhGLP2*	1	1500	795	3	264	28.03845	9.12	PlasmaMembrane
AH01G21370.1	*AhGLP1-2*	*AhGLP3*	1	552	552	1	183	19.73137	5.16	Extracellular
AH01G21380.1	*AhGLP1-3*	*AhGLP4*	1	636	636	1	211	22.83725	6.4	PlasmaMembrane
AH01G21390.1	*AhGLP1-4*	*AhGLP5*	1	582	582	1	193	21.04495	5.22	Extracellular
AH01G27580.1	*AhGLP1-5*	*AhGLP6*	1	1076	675	2	224	24.28661	6.41	Extracellular
AH02G08870.1	*AhGLP2-1*	*AhGLP7*	2	3066	1908	7	635	72.95963	5.32	Nuclear
AH02G09900.1	*AhGLP2-2*	*AhGLP8*	2	3077	1101	8	366	39.54861	5.13	Cytoplasmic
AH02G15120.1	*AhGLP2-3*	*AhGLP9*	2	2546	1377	4	458	51.4487	5.92	Chloroplast
AH02G16210.1	*AhGLP2-4*	*AhGLP10*	2	522	522	1	173	18.74532	6.17	Extracellular
AH03G01760.1	*AhGLP3-1*	*AhGLP11*	3	2876	1806	5	601	67.41861	5.49	Cytoplasmic
AH03G24080.1	*AhGLP3-2*	*AhGLP12*	3	636	636	1	211	21.91025	6.25	Extracellular/PlasmaMembrane
AH03G27670.1	*AhGLP3-3*	*AhGLP13*	3	996	660	2	219	22.91238	8.85	Mitochondrial
AH03G44290.1	*AhGLP3-4*	*AhGLP14*	3	2424	1074	3	357	38.46907	5.45	Cytoplasmic/Chloroplast
AH04G09310.1	*AhGLP4-1*	*AhGLP15*	4	757	660	2	219	23.16438	6.03	Extracellular
AH05G06560.1	*AhGLP5-1*	*AhGLP16*	5	2232	1947	5	648	76.1751	5.34	Nuclear
AH06G05360.1	*AhGLP6-1*	*AhGLP17*	6	675	675	1	224	23.44566	5.9	Chloroplast
AH06G05510.1	*AhGLP6-2*	*AhGLP18*	6	957	660	2	219	22.97853	9.06	Mitochondrial
AH06G07300.1	*AhGLP6-3*	*AhGLP19*	6	3799	1368	4	455	51.1761	5.32	Extracellular
AH06G07310.1	*AhGLP6-4*	*AhGLP20*	6	2513	1464	5	487	55.59726	5.67	Nuclear
AH06G07320.1	*AhGLP6-5*	*AhGLP21*	6	2518	1464	5	487	55.59726	5.67	Nuclear
AH06G07330.1	*AhGLP6-6*	*AhGLP22*	6	3672	1224	4	407	45.87418	5.44	Extracellular
AH06G07340.1	*AhGLP6-7*	*AhGLP23*	6	1122	1038	2	345	39.6365	5.26	Nuclear
AH06G07350.1	*AhGLP6-8*	*AhGLP24*	6	2536	1383	4	460	50.76401	5.65	Chloroplast
AH06G07560.1	*AhGLP6-9*	*AhGLP25*	6	1732	1281	4	426	46.82439	5.28	Cytoplasmic
AH06G08990.1	*AhGLP6-10*	*AhGLP26*	6	21973	669	3	222	24.49791	9.36	Extracellular/PlasmaMembrane
AH06G09010.1	*AhGLP6-11*	*AhGLP27*	6	819	591	3	196	21.17122	6.71	Extracellular
AH06G09020.1	*AhGLP6-12*	*AhGLP28*	6	754	654	2	217	23.48304	6.9	Extracellular
AH06G09030.1	*AhGLP6-13*	*AhGLP29*	6	99291	747	3	248	27.03766	9.42	Extracellular/PlasmaMembrane
AH06G10010.1	*AhGLP6-14*	*AhGLP30*	6	1876	1593	4	530	60.51845	5.41	Nuclear
AH06G11370.1	*AhGLP6-15*	*AhGLP31*	6	1681	1401	5	466	52.96232	6.49	Cytoplasmic/Nuclear
AH06G14500.1	*AhGLP6-16*	*AhGLP32*	6	748	654	2	217	23.53536	9.25	Extracellular
AH08G12440.1	*AhGLP8-1*	*AhGLP33*	8	1834	1071	3	356	38.25788	5.78	Chloroplast
AH08G13790.1	*AhGLP8-2*	*AhGLP34*	8	2208	1440	5	479	53.91077	5.55	Cytoplasmic
AH08G15640.1	*AhGLP8-3*	*AhGLP35*	8	552	552	1	183	19.73536	5.16	Extracellular
AH08G15650.1	*AhGLP8-4*	*AhGLP36*	8	636	636	1	211	22.83016	5.75	PlasmaMembrane
AH08G23470.1	*AhGLP8-5*	*AhGLP37*	8	2403	738	2	245	27.08769	5.51	PlasmaMembrane/Mitochondrial
AH09G09070.1	*AhGLP9-1*	*AhGLP38*	9	666	666	1	221	23.5081	7.78	Extracellular/PlasmaMembrane
AH09G09090.1	*AhGLP9-2*	*AhGLP39*	9	627	627	1	208	21.809	6.03	Extracellular/PlasmaMembrane
AH09G09100.1	*AhGLP9-3*	*AhGLP40*	9	630	630	1	209	21.59614	6.49	PlasmaMembrane
AH09G10430.1	*AhGLP9-4*	*AhGLP41*	9	669	669	1	222	23.59424	6.96	Extracellular/PlasmaMembrane
AH09G11970.1	*AhGLP9-5*	*AhGLP42*	9	7889	663	2	220	23.24688	7.74	Chloroplast
AH09G26180.1	*AhGLP9-6*	*AhGLP43*	9	2065	1845	4	614	70.28306	6.43	Nuclear
AH09G29830.1	*AhGLP9-7*	*AhGLP44*	9	627	627	1	208	22.56223	7.74	Extracellular/Chloroplast
AH10G19070.1	*AhGLP10-1*	*AhGLP45*	10	672	672	1	223	24.39974	5.45	PlasmaMembrane
AH11G30090.1	*AhGLP11-1*	*AhGLP46*	11	1175	675	2	224	24.29472	6.89	Extracellular
AH12G10310.1	*AhGLP12-1*	*AhGLP47*	12	3406	1863	7	620	71.63151	5.81	Nuclear
AH12G18420.1	*AhGLP12-2*	*AhGLP48*	12	3211	1380	4	459	51.62868	5.81	Cytoplasmic
AH12G19300.1	*AhGLP12-3*	*AhGLP49*	12	1049	675	2	224	24.19668	6.28	Extracellular
AH12G35470.1	*AhGLP12-4*	*AhGLP50*	12	558	558	1	185	19.87863	6.37	Cytoplasmic/Chloroplast
AH13G03640.1	*AhGLP13-1*	*AhGLP51*	13	2458	1809	5	602	67.70997	5.84	Cytoplasmic/Chloroplast
AH13G27080.1	*AhGLP13-2*	*AhGLP52*	13	636	636	1	211	21.8822	6.25	Extracellular/PlasmaMembrane
AH13G33560.1	*AhGLP13-3*	*AhGLP53*	13	558	558	1	185	19.97288	6.89	Extracellular
AH13G46980.1	*AhGLP13-4*	*AhGLP54*	13	2475	1074	3	357	38.46209	5.46	PlasmaMembrane/Chloroplast
AH13G59860.1	*AhGLP13-5*	*AhGLP55*	13	1554	849	3	282	29.73555	9.4	PlasmaMembrane
AH14G08910.1	*AhGLP14*	*AhGLP56*	14	758	660	2	219	23.31455	6.13	Extracellular
AH15G02710.1	*AhGLP15-1*	*AhGLP57*	15	2289	1980	5	659	77.15899	5.17	Nuclear
AH15G11750.1	*AhGLP15-2*	*AhGLP58*	15	675	675	1	224	24.299	8.71	Extracellular/PlasmaMembrane
AH16G01900.1	*AhGLP16-1*	*AhGLP59*	16	2417	1401	5	466	53.01923	6.18	Cytoplasmic/Nuclear
AH16G08990.1	*AhGLP16-2*	*AhGLP60*	16	675	675	1	224	23.56089	5.61	Extracellular/Chloroplast
AH16G09160.1	*AhGLP16-3*	*AhGLP61*	16	963	660	2	219	22.92144	8.85	Mitochondrial
AH16G12850.1	*AhGLP16-4*	*AhGLP62*	16	71124	627	3	208	22.64782	8.93	Extracellular
AH16G12950.1	*AhGLP16-5*	*AhGLP63*	16	748	654	2	217	23.33887	6.9	Extracellular
AH16G14260.1	*AhGLP16-6*	*AhGLP64*	16	3872	2145	10	714	81.39978	6.19	PlasmaMembrane/Nuclear
AH16G14850.1	*AhGLP16-7*	*AhGLP65*	16	1583	1281	4	426	46.82431	5.16	Cytoplasmic
AH16G15180.1	*AhGLP16-8*	*AhGLP66*	16	2420	1380	4	459	50.48476	5.8	Chloroplast
AH16G15190.1	*AhGLP16-9*	*AhGLP67*	16	2210	1539	4	512	58.26331	5.41	Nuclear
AH16G15220.1	*AhGLP16-10*	*AhGLP68*	16	2307	1716	4	571	65.21118	5.57	Extracellular/PlasmaMembrane
AH16G15240.1	*AhGLP16-11*	*AhGLP69*	16	2312	1716	4	571	65.21118	5.57	Extracellular/PlasmaMembrane
AH16G15250.1	*AhGLP16-12*	*AhGLP70*	16	3515	1455	4	484	54.55463	5.4	Extracellular
AH18G05950.1	*AhGLP18-1*	*AhGLP71*	18	552	552	1	183	19.74939	5.16	Extracellular
AH18G05960.1	*AhGLP18-2*	*AhGLP72*	18	636	636	1	211	22.75416	6.04	PlasmaMembrane
AH18G05970.1	*AhGLP18-3*	*AhGLP73*	18	582	582	1	193	20.91179	5.01	Extracellular
AH18G14270.1	*AhGLP18-4*	*AhGLP74*	18	1830	1071	3	356	38.24582	5.78	Chloroplast
AH18G27460.1	*AhGLP18-5*	*AhGLP75*	18	678	678	1	225	25.06955	5.7	Extracellular/PlasmaMembrane
AH19G12060.1	*AhGLP19-1*	*AhGLP76*	19	663	663	1	220	23.461	7.79	Extracellular/PlasmaMembrane
AH19G12070.1	*AhGLP19-2*	*AhGLP77*	19	627	627	1	208	21.79202	6.89	Extracellular/PlasmaMembrane
AH19G12080.1	*AhGLP19-3*	*AhGLP78*	19	630	630	1	209	21.55416	6.95	PlasmaMembrane
AH19G13680.1	*AhGLP19-4*	*AhGLP79*	19	669	669	1	222	23.61723	6.17	PlasmaMembrane
AH19G15620.1	*AhGLP19-5*	*AhGLP80*	19	2366	666	2	221	23.24289	7.75	Extracellular/PlasmaMembrane/Chloroplast
AH19G38160.1	*AhGLP19-6*	*AhGLP81*	19	627	627	1	208	22.56219	6.83	Chloroplast
AH19G41920.1	*AhGLP19-7*	*AhGLP82*	19	2275	1881	4	626	71.34521	6.62	Nuclear
AH20G11080.1	*AhGLP20-1*	*AhGLP83*	20	3466	1395	5	464	49.72532	8.77	PlasmaMembrane
AH20G25130.1	*AhGLP20-2*	*AhGLP84*	20	672	672	1	223	24.33964	5.45	PlasmaMembrane

Chr, Chromosome; MW, molecular weight; pI, theoretical isoelectric point; * GLPs were renamed to include the chromosome numbers, ** GLPs were renamed according to their genomic positions without indicating the chromosome numbers.

### 3.2 Gene structure, conserved motifs, and phylogenetic analysis

**Figure 2:**
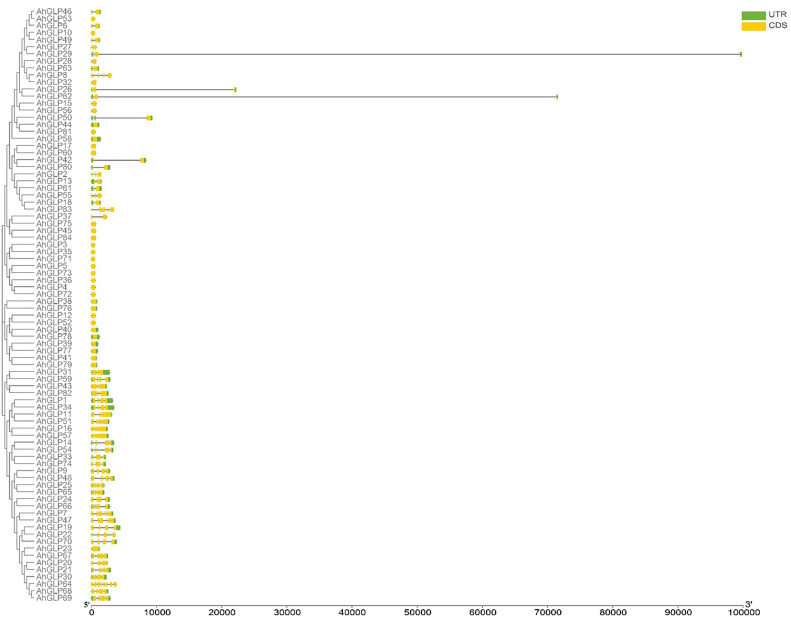
Gene structure (exons-introns distribution) of *Arachis hypogaea* Germin-like protein (*AhGLPs*) genes. Most *AhGLPs* are composed of a single exon, while the maximum number of exons is 10 (*AhGLP64*). *AhAGLP26*, *AhGLP29*, and *AhGLP62* possessed very large intron sequences.

**Figure 3:**
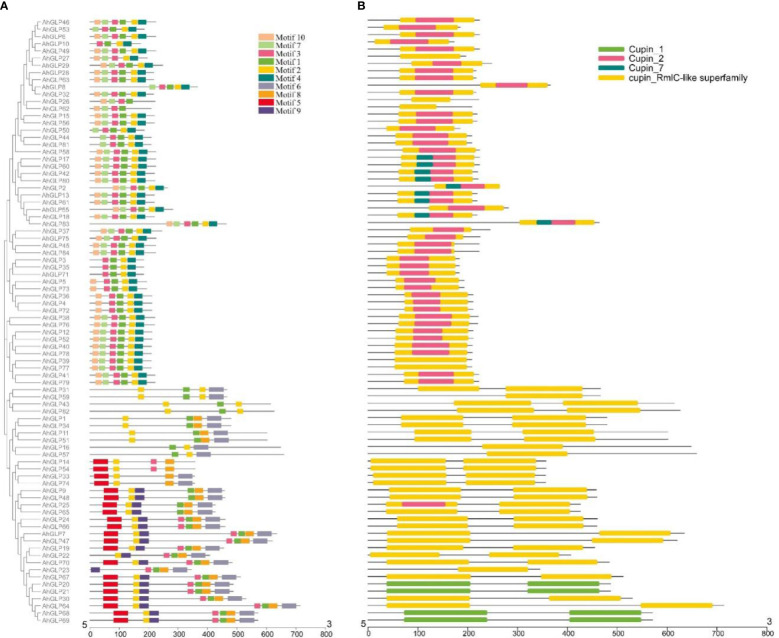
Conserved motifs distribution patterns **(A)** and presence of cupin domains **(B)** in *Arachis hypogaea* Germin-like proteins (*AhGLPs*). Commonly shared motifs among genes tend to cluster in the same groups, referring to their similar functions.

Several *GLPs* of cultivated peanut were composed of a single exon (29 out of 84), while the maximum number of exons was 10 (*AhGLP64*). Gene length variations of *AhGLPs* were in accordance with its diploid parents, as the smallest *GLPs* in diploid progenitors were *AdGLP3* (552 bp), *AdGLP34* (552 bp) (*A. duranensis*), and *AiGLP32* (459 bp), *AiGLP28* (552 bp) (*A. ipaensis*), similarly the smallest *GLPs* in cultivated peanut were *AhGLP3*, *AhGLP10*, *AhGLP35*, and *AhGLP71* with the genomic length of 552 bp. But few genes in cultivated peanut possessed extraordinary long genomic sequences, for example, *AhGLP62* with a genomic length of 71124 bp and *AhGLP29* with a genomic length of 99291 bp (**Figure 2**). Scanning of protein sequences at the MEME server identified many common and unique motifs. Commonly shared motifs among genes tend to cluster in the same groups, referring to their similar functions. The length of motifs was also different, i.e., the maximum motif length was 50 amino acid residues (5th and 6th motifs), and the minimum motif length was 21 amino acid residues for the 2^nd^, 3^rd^, 7^th^, and 10^th^ motifs (**Supplementary Table 3**). Conserved motifs distribution patterns of *AhGLPs* are shown in **Figure 3** **A**, and cupin domain locations are shown in **Figure 3** **B**. The gene structure and motif distribution patterns of *AhGLPs* are in agreement with *A. duranensis* and *A. ipaensis GPLs* (**Supplementary Figures 3, 4**, **Supplementary Tables 4, 5**).

**Figure 4:**
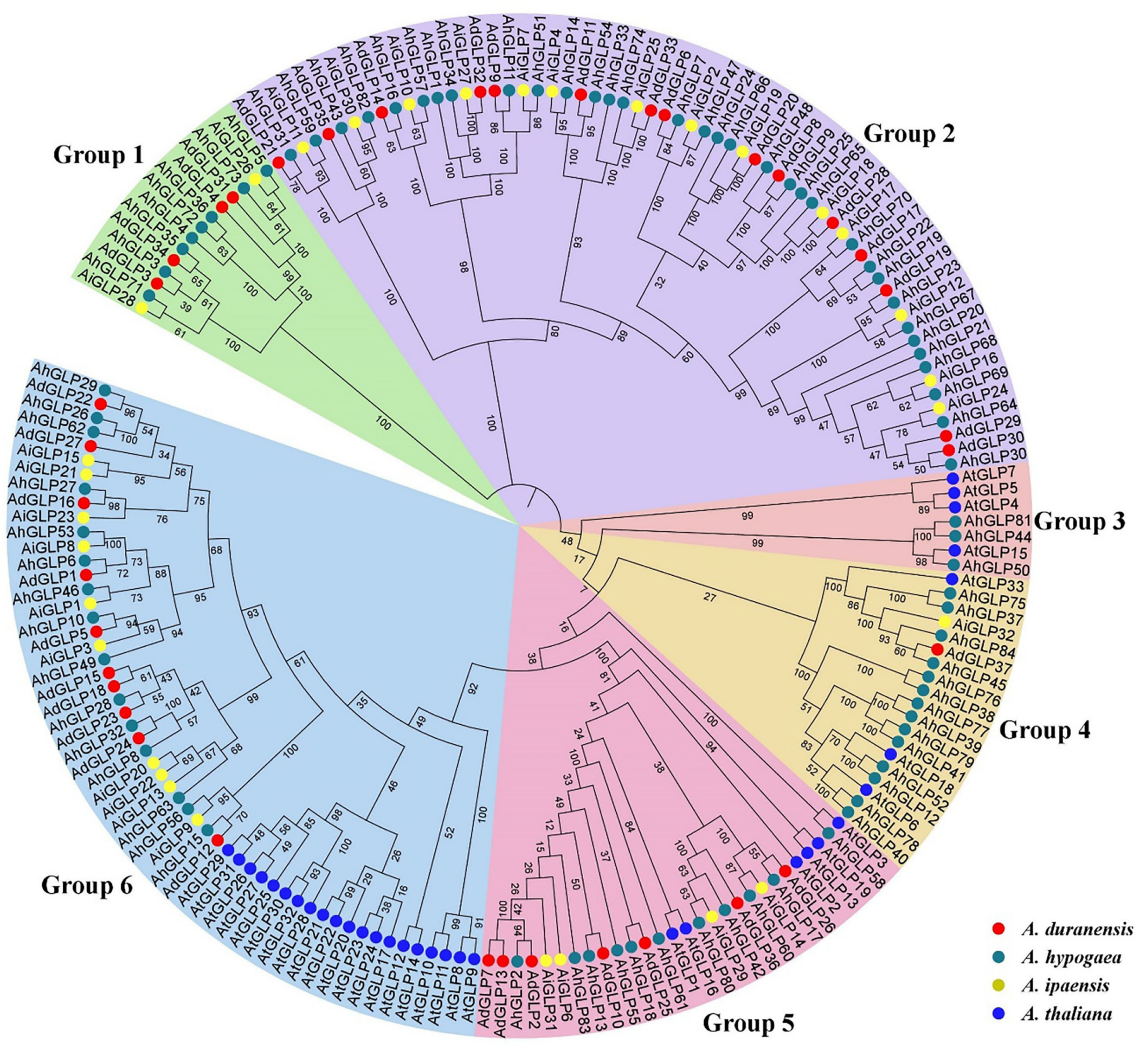
Phylogenetic relationships of Germin-like proteins of *A. hypogea*, *A. duranensis*, *A. ipaensis*, and *A. thaliana*. The phylogenetic tree classified peanut GLPs into six groups, while *AtGLPs* were restricted to only four groups. Germin-like proteins of cultivated peanut are phylogenetically closer to its wild parents as compared to *Arabidopsis*.

The evolutionary relationships of germin-like proteins of peanuts and *Arabidopsis* were elucidated by constructing the phylogenetic tree of their protein sequences. The higher number of GLPs present in cultivated peanut than in its diploid parents and *Arabidopsis* indicates a higher evolutionary rate of *AhGLPs*. Previous studies classified *GLPs* into six phylogenetic groups in barley, wheat, soybean, and rice (Lu et al., 2010; Barman and Banerjee, 2015; Li et al., 2016; Yuan et al., 2021), and the phylogenetic tree also divided peanut *GLPs* into six phylogenetic groups. All proteins of *Arabidopsis* were restricted to four groups such as groups 3, 4, 5, and 6 (**Figure 4**). *GLPs* in the first and second groups were only from peanut species, indicating that cultivated peanut was closer to its wild progenitors compared with *Arabidopsis*.

### 3.3 Identification of orthologous gene clusters

**Figure 5:**
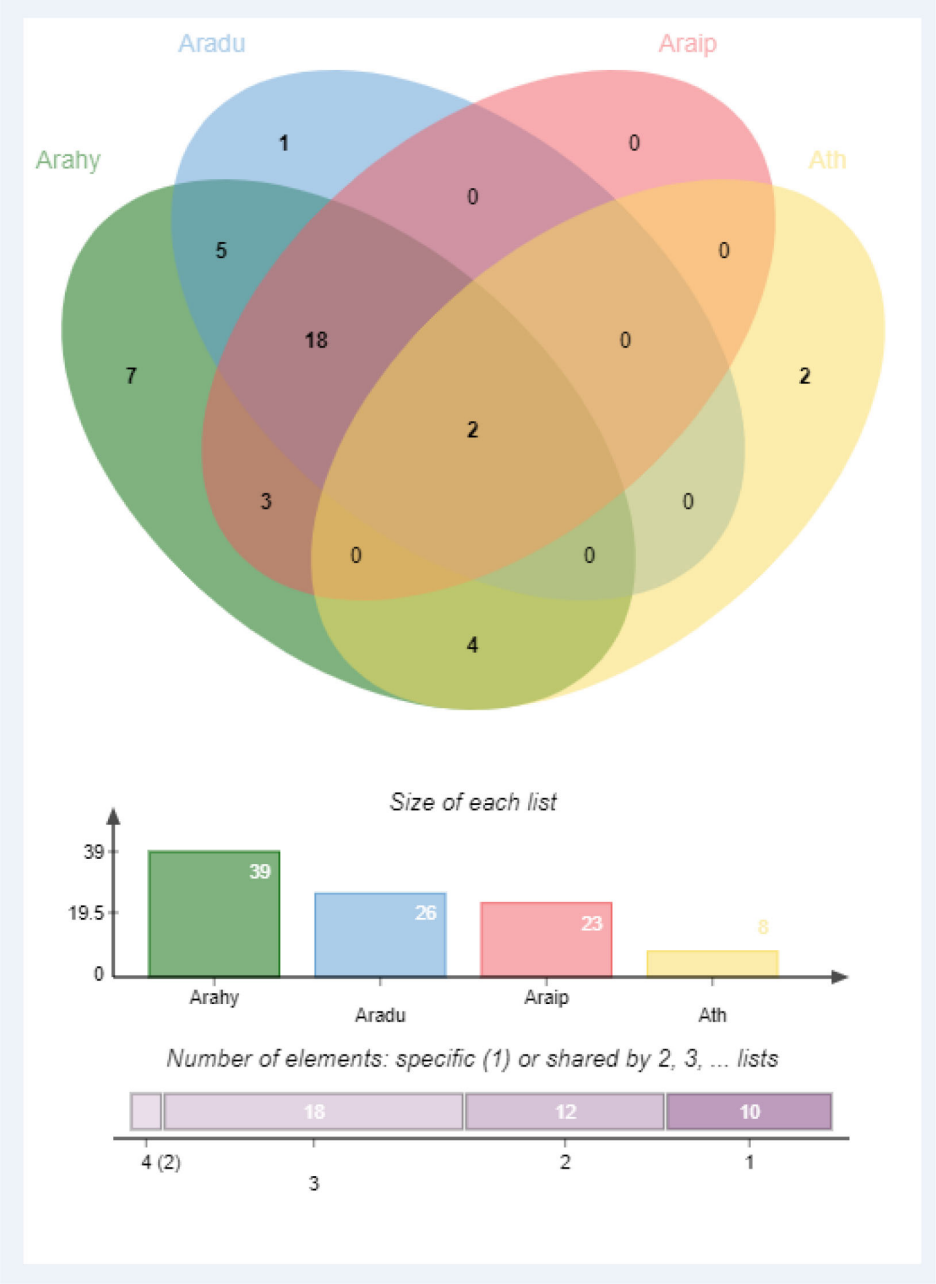
Orthologous genes’ clusters among *A. hypogea*, *A. duranensis*, *A. ipaensis*, and *A. thaliana*. *A. hypogea* recorded maximum clustered. Eighteen gene clusters are solely composed of GLPs found in peanut diploid and tetraploid species, indicating that polyploidization has evolved new peanut-specific orthologous GLP gene clusters.

Identification of orthologous genes’ clusters is important to evaluate the polyploidization events during the evolutionary process of a gene family. The relative assessment was established to detect the orthologous gene clusters across *A. hypogea*, *A. duranensis*, *A. ipaensis*, and *A. thaliana*. **Figure 5** elaborates the identified gene clusters and their overlapping regions. *A. hypogea* recorded maximum clusters followed by *A. duranensis*, *A. ipaensis*, and *A. thaliana*. Results showed that 18 gene clusters are solely composed of GLPs found in peanut species (diploid and tetraploid), which indicated that polyploidization had evolved new peanut-specific orthologous *GLP* gene clusters. We also constructed orthologous gene clusters among three peanut species (**Supplementary Figure 5**). Comparatively, 60, 52, 13, and 55 orthologous *GLPs* were found in *A. hypogea*, *A. duranensis*, *A. ipaensis*, and *A. thaliana*, respectively (Supplementary File 2). Thirty-six in-paralogous genes were identified in *A. hypogea*, eight and four in-paralogous genes were identified in *A. duranensis* and *A. ipaensis*, while 178 in-paralogous genes were found in *A. thaliana*. Surprisingly 12, 5, and 4 singletons were also found in *A. hypogea*, *A. duranensis*, and *A. ipaensis*, respectively. Results demonstrated that identified orthologous genes decrease with increased phylogenetic distances.

### 3.4 Analysis of *cis*-regulatory elements

**Figure 6:**
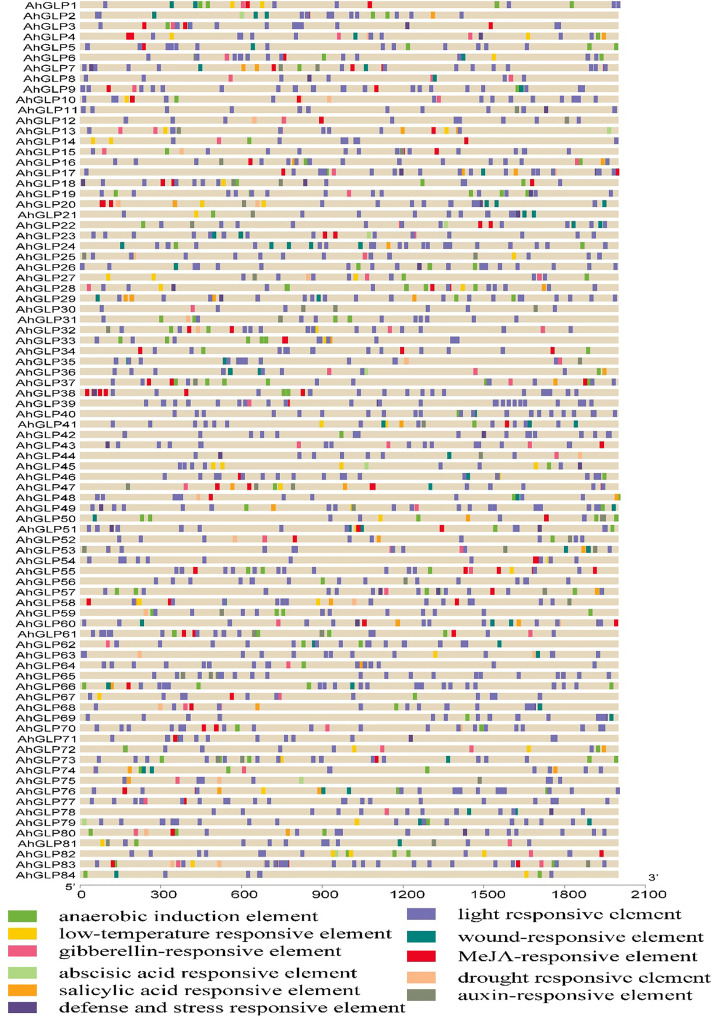
*Cis*-regulatory elements of *AhGLP* promoters. *Cis*-elements analysis revealed important elements responsive to light, hormones, growth and development, and stress responsiveness.

**Figure 7:**
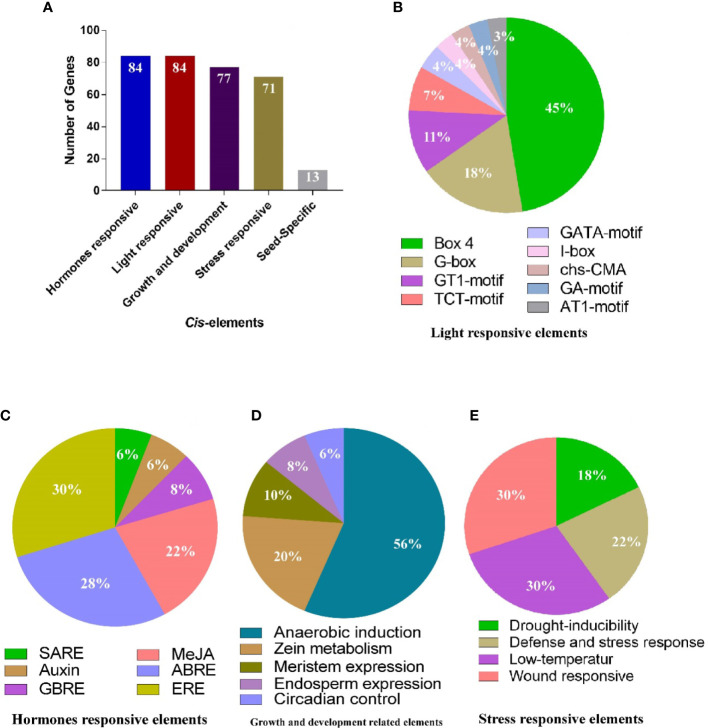
*Cis*-regulatory elements of *AhGLP* promoters. **(A)** the number of genes in different categories of elements. **(B)** composition of light-responsive, **(C)** hormone-responsive, **(D)** growth and development responsive, and **(E)** stress-responsive factors.

For functional genomics studies, researchers must explore the genomic regions to identify the transcription factor binding sites or groups of sites constituting the *cis*-regulatory elements (Raza et al., 2021b). To predict the gene functions and their regulatory patterns, we searched the *cis*-regulatory elements of *AhGLPs* promoter regions. The *cis*-elements prediction results showed that in addition to core promoter elements (TATA-Box, CAAT-Box), a large number of important elements were also present (**Figure 6**). We classified these *cis*-regulatory elements into four groups according to their functions: ‘light-responsive’, ‘hormones responsive’, ‘growth and development-related’, and ‘stress-related’ elements (**Figure 7**). All 84 *AhGLPs* were enriched with hormones- and light-responsive elements, 77 genes were enriched with growth and development-related elements, and 71 genes were enriched with stress-responsive elements (**Figure 7** **A**). Thirteen genes were also enriched in RY-element, which is responsible for seed-specific regulation (**Figure 7** **A**) (Stålberg et al., 1993).

Light responsive elements mainly include Box-4, G-box, GT1-motif, GATA-motif, TCT-motif, GA-motif, chs-CMA element, I-box, and AT-1 motif (**Figure 7** **B**). Other light-responsive elements include 3-AF1 binding site, ATC-motif, AE-box, MRE element, Box II, CAG-motif, CGTCA-motif ATCT-motif, Gap-box, ACE element, TCCC-motif, GTGGC-motif, LAMP-element, LS7 element, and Sp1 element, were also present. Hormones responsive class includes abscisic acid-responsive (ABRE), auxin-responsive (AuxRE, AuxRR-core, CGTCA-motif, TGA-box), gibberellins responsive (P-box, GARE motif, TATC-box), methyl jasmonate responsive (CGTCA-motif, TGACG-motif), salicylic acid-responsive (SARE, TCA-element), and ethylene-responsive (ERE) elements (**Figure 7** **C**). Growth and development category contained anaerobic induction responsive (ARE), meristem expression responsive (CAT-box), endosperm expression related (GCN4-motif, AACA-motif), circadian control (CAAAGATATC), and zein metabolism-related (O2-site) elements (**Figure 7** **D**). The stress-responsive class further includes defense and stress response (TC-rich repeats), drought-responsive (MBS), low-temperature responsive (LTR), and wound-related (WUN-motif) elements (**Figure 7** **E**).

### 3.5 Genome-wide identification of miRNAs targeting the *AhGLPs*

**Figure 8:**
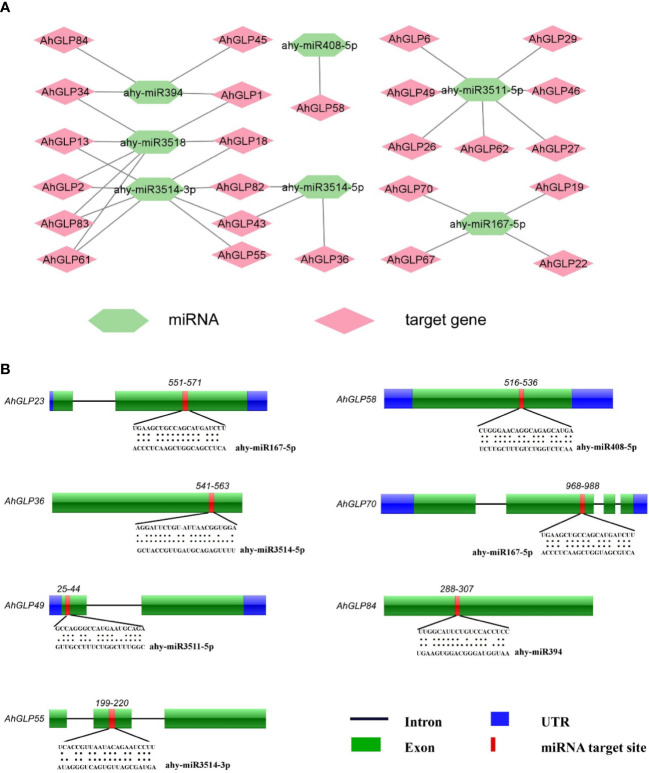
Predicted miRNAs targeting *AhGLPs*. **(A)** In total, 7 miRNAs were predicted targeting 25 *Arachis hypogea* Germin-like protein gens. **(B)** Schematic diagram of miRNAs target sites in some *AhGLPs*.

During the past decade, non-coding miRNAs have emerged as key regulators of post-transcriptional gene regulation (Chen et al., 2019; Raza et al., 2021a). Their roles have been worked out against many biotic and abiotic stresses (Sun et al., 2015; Ding et al., 2017). To better understand miRNAs modulating the post-transcriptional regulation of *AhGLPs*, the coding sequences of 84 *AhGLPs* were scanned at the psRNATarget database (https://www.zhaolab.org/psRNATarget/analysis?function=2) against the published *A. hypogaea* miRNAs. Scanning results predicted seven miRNAs from six different families targeting 25 peanut germin-like proteins (**Supplementary Table 6**). The miRNA ‘ahy-miR3514-3p’ targeted eight *AhGLPs* (the highest number of *AhGLPs* targeted by any miRNA). While ‘miRNA ahy-miR3511-5p’ and ‘ahy-miR3518’ targeted seven *AhGLPs* each. The miRNA ‘ahy-miR167-5p’ and ‘ahy-miR394’ targeted four *AhGLPs* each. The ‘ahy-miR3514-5p’ targeted three *AhGLPs* and ‘ahy-miR408-5p’ targeted only one peanut *GLP* (**Figure 8** **A**). The germin-like protein genes *AhGLP1*, *AhGLP2*, *AhGLP13*, *AhLP18*, *AhGLP34*, *ahGLP43*, *AhGLP61*, *AhGLP82*, and *AhGLP83* each were targeted by more than one miRNAs (two) while the remaining genes were targeted by one miRNA. Some of the miRNAs target sites are shown in **Figure 8** **B**. Functional validation of the expression level of these miRNAs and their role in gene regulation in the peanut genome are potential future research dimensions.

### 3.6 Gene duplication and synteny analysis

**Figure 9:**
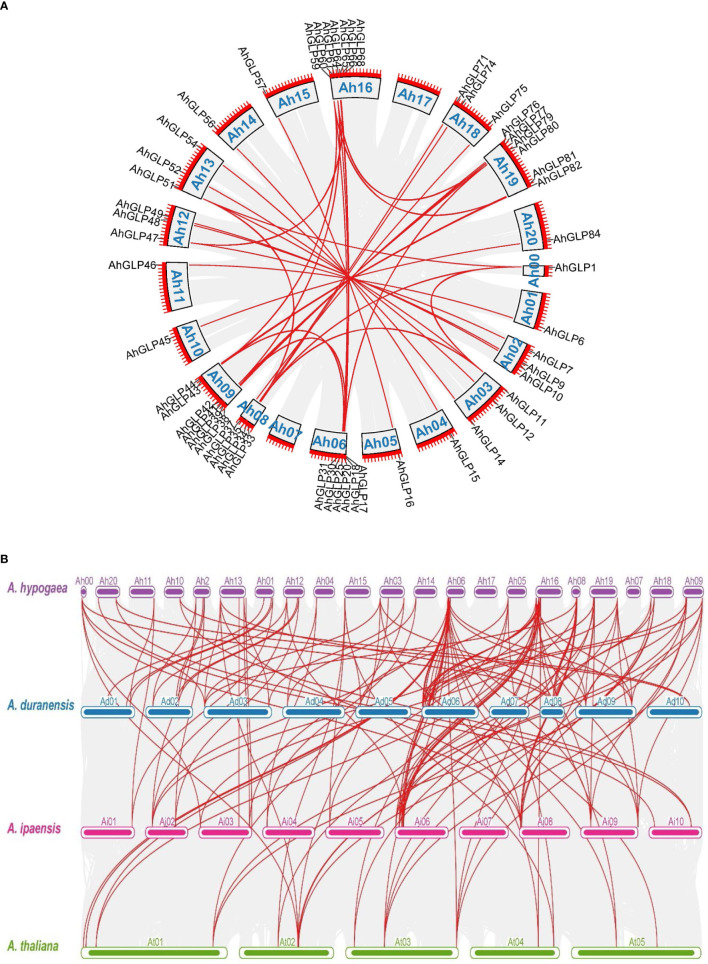
**(A)** Schematic diagram of gene duplication in *AhGLPs*, red lines indicate the duplicated gene pairs and grey lines in the background represent the duplicated regions among different chromosomes. **(B)** Synteny analysis among *A hypogea*, *A duranensis*, *A ipaensis*, and *A thaliana*. Synteny analysis showed key evolutionary relationships of *GLPs* in diploid and tetraploid peanut species. *AhGLPs* possessed highly conserved syntenic relationships with other peanut species as compared to *Arabidopsis*.

Gene duplication (segmental and tandem duplication) is a major force behind genome evolution (Su et al., 2021). So, gene duplication events for *A. hypogea* were evaluated. Mainly segmental gene duplication was found to be responsible for deriving the genome evolution (**Figure 9** **A**). Out of 84 *AhGLPs*, 38 were duplicated gene pairs (**Table 2**). The synonymous substitution rates Ks and non-synonymous substitution rates Ka were calculated by the simple Ka/Ks calculator. For each duplicated gene pair, evolutionary rates (Ka/Ks ratio) were calculated. The Ka/Ks=1 was considered neutral selection pressure, while Ka/Ks>1 was regarded as positive selection pressure, and Ka/Ks<1 was considered purifying selection pressure (Yang and Bielawski, 2000). Mainly purifying selection pressure was involved in gene duplication (**Table 2**). The expected divergence time ‘T’ of duplicated gene pairs was calculated as ‘T=Ks/2r’, where the exchange rate coefficient ‘r’ for the peanut is 8.12×10^-9^ (Bertioli et al., 2016). The divergence time varied from 0.43 MYA (million years ago) for gene pair *AhGLP44:AhGLP81* to 166.24 MYA for *AhGLP47:AhGLP66* (**Table 2**).

**Table 2:** Identification of duplicated gene pairs among *AhGLPs* and their expected divergence time.

Seq_1	Seq_2	Ka	Ks	Ka/Ks	Selection pressure	Time
*AhGLP6*	*AhGLP46*	0.013739344	0.039012	0.35218211	Purifying	2.402219
*AhGLP7*	*AhGLP47*	0.013641635	0.035426	0.385077233	Purifying	2.181386
*AhGLP9*	*AhGLP48*	0.01468132	0.045385	0.323487132	Purifying	2.794616
*AhGLP10*	*AhGLP49*	0.011416711	0.037876	0.301421677	Purifying	2.332279
*AhGLP11*	*AhGLP34*	0.353186717	1.07438	0.328735256	Purifying	66.15643
*AhGLP11*	*AhGLP51*	0.01425221	0.070853	0.201152095	Purifying	4.362864
*AhGLP11*	*AhGLP1*	0.353970053	1.095105	0.323229261	Purifying	67.43259
*AhGLP12*	*AhGLP52*	0.010578455	0.045966	0.23013529	Purifying	2.830434
*AhGLP14*	*AhGLP54*	0.008575022	0.036893	0.232431507	Purifying	2.271717
*AhGLP15*	*AhGLP56*	0.012255592	0.024886	0.492476415	Purifying	1.532367
*AhGLP16*	*AhGLP57*	0.014115691	0.040456	0.348911167	Purifying	2.491158
*AhGLP17*	*AhGLP42*	0.167084236	0.860359	0.194202981	Purifying	52.97776
*AhGLP17*	*AhGLP60*	0.01988188	0.050975	0.39003176	Purifying	3.138856
*AhGLP17*	*AhGLP80*	0.172570105	0.861782	0.200247897	Purifying	53.06542
*AhGLP18*	*AhGLP61*	0.006120401	0.018425	0.33218507	Purifying	1.134524
*AhGLP20*	*AhGLP68*	0.021532579	0.087383	0.246415553	Purifying	5.380739
*AhGLP25*	*AhGLP65*	0.008268818	0.043857	0.188538925	Purifying	2.700576
*AhGLP30*	*AhGLP64*	0.023549167	0.069371	0.339467272	Purifying	4.27161
*AhGLP31*	*AhGLP43*	0.670764421	NaN	NaN	NaN	NaN
*AhGLP31*	*AhGLP59*	0.020603762	0.04238	0.486172255	Purifying	2.609578
*AhGLP33*	*AhGLP74*	0.00123839	0.035463	0.034920689	Purifying	2.183679
*AhGLP34*	*AhGLP51*	0.331929492	0.963077	0.344655135	Purifying	59.30278
*AhGLP34*	*AhGLP1*	0.008010757	0.013122	0.610470872	Purifying	0.808021
*AhGLP35*	*AhGLP71*	0.012005058	0.023593	0.50883912	Purifying	1.452773
*AhGLP37*	*AhGLP75*	0.01945354	0.014389	1.351979572	Positive	0.886018
*AhGLP38*	*AhGLP76*	0.010040311	0.045468	0.220819205	Purifying	2.799782
*AhGLP39*	*AhGLP77*	0.010703	0.040143	0.266619435	Purifying	2.471882
*AhGLP41*	*AhGLP79*	0.015971324	0.019018	0.839780448	Purifying	1.171087
*AhGLP42*	*AhGLP60*	0.171823216	0.849225	0.202329493	Purifying	52.29217
*AhGLP42*	*AhGLP80*	0.012216083	0.050175	0.24346735	Purifying	3.089621
*AhGLP43*	*AhGLP59*	0.671654809	NaN	NaN	NaN	NaN
*AhGLP43*	*AhGLP82*	0.013103863	0.059803	0.21911805	Purifying	3.682436
*AhGLP44*	*AhGLP81*	0.004173924	0.007001	0.596171122	Purifying	0.431109
*AhGLP45*	*AhGLP84*	0.005859405	0.019609	0.298812623	Purifying	1.207448
*AhGLP47*	*AhGLP66*	0.471519438	2.699874	0.174644954	Purifying	166.2484
*AhGLP51*	*AhGLP1*	0.329288271	0.985559	0.334113067	Purifying	60.68715
*AhGLP59*	*AhGLP82*	0.707166033	NaN	NaN	NaN	NaN
*AhGLP60*	*AhGLP80*	0.178586333	0.841147	0.212312751	Purifying	51.79479

Time represents expected divergence time as million years ago (MYA).

Comparative synteny analysis among *A. hypogaea*, *A. duranensis*, *A. ipaensis*, and *A. thaliana* represented remarkable evolutionary, duplication, expression, and functional relationships. Mainly *AhGLPs* showed significant syntenic relationships with its wild progenitors and *Arabidopsis*; however, the syntenic relationships of *A. hypogaea* were closer to its wild parents than *Arabidopsis*. Total 69 syntenic relationships of *A. hypogaea* were found in the genome of *A. duranensis*, and 57 syntenic relationships of *AhGLPs* were found in the genome of *A. ipaensis*. In contrast, 31 syntenic relationships were found among *AhGLPs* and *AtGLPs* (Supplementary File 3). The synteny analysis results showed that *A. hypogaea* is closer to its wild parents than *Arabidopsis*. The syntenic relations of *A. hypogaea*, *A. duranensis*, *A. ipaensis*, and *A. thaliana* are shown in **Figure 9** **B**.

### 3.7 Prediction of protein-protein interaction network

**Figure 10:**
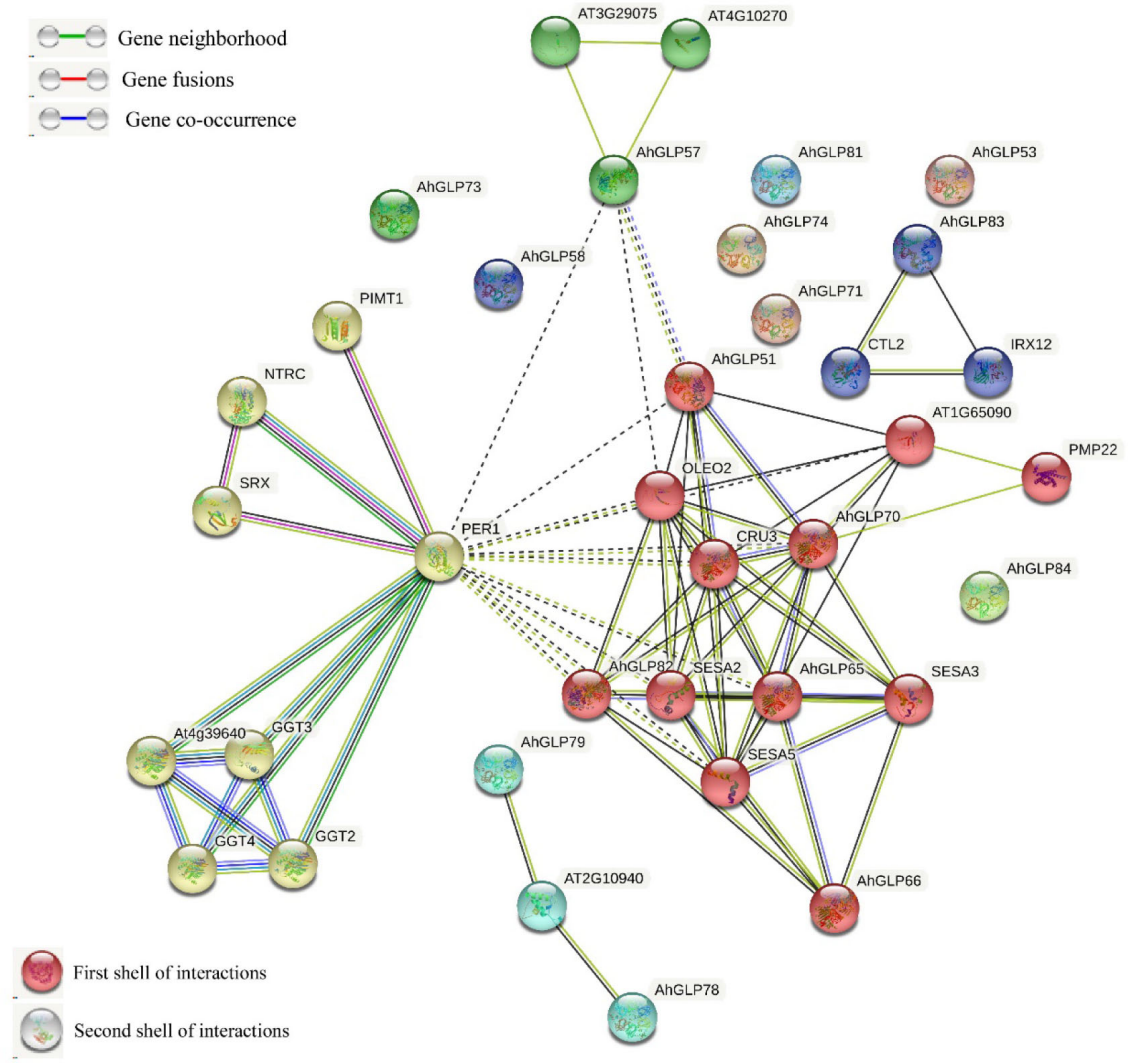
The predicted protein-protein interaction network of *AhGLPs* using the STRING database. Putative protein functions of *AhGLPs* are predicted on well-studied GLP orthologues in *Arabidopsis*.

The Functions of *AhGLPs* could be speculated on the basis of well-studied *Arabidopsis GLPs*. The protein-protein interaction network analysis of cultivated peanut GLPs were performed to understand the functions of GLP proteins on the basis of their orthologues in *Arabidopsis*. Protein interaction network prediction showed that *AhGLPs* have functions related to cysteine peroxidation 1 (PER1), contributing to inhibition of germination under stressed conditions (**Figure 10**). *AhGLP82* has those related to oleosin2 (OLEO2) found in oil bodies and plays roles in freeze tolerance in seeds, and CUR3, a seed storage protein, mainly plays a role in response to ABA stress. *AhGLP82* also possesses functions related to seed storage as of SESA2, SESA3, and SESA5. Other *AhGLPs* also had similar those and those related to proximal membrane proteins (*AhGLP65* and *AhGLP70*). *AhGLP83* was predicted to interact with *Arabidopsis* Chitinase-like protein (preventing the lignin accumulation in hypocotyls CTL2) and Laccase/diphenol oxidase family protein IRX12 (cell wall biosynthesis-related functions). Multiple sequence search method based on the scoring and integration of known and predicted associations results in comprehensive networks showed that some proteins, including *AhGLP53*, *AhGLP58*, *AhGLP71*, *AhGLP73*, *AhGLP74*, *AhGLP71*, and *AhGLP84*, did not show any interaction.

### 3.8 Functional annotation analysis of AhGLPs

**Figure 11:**
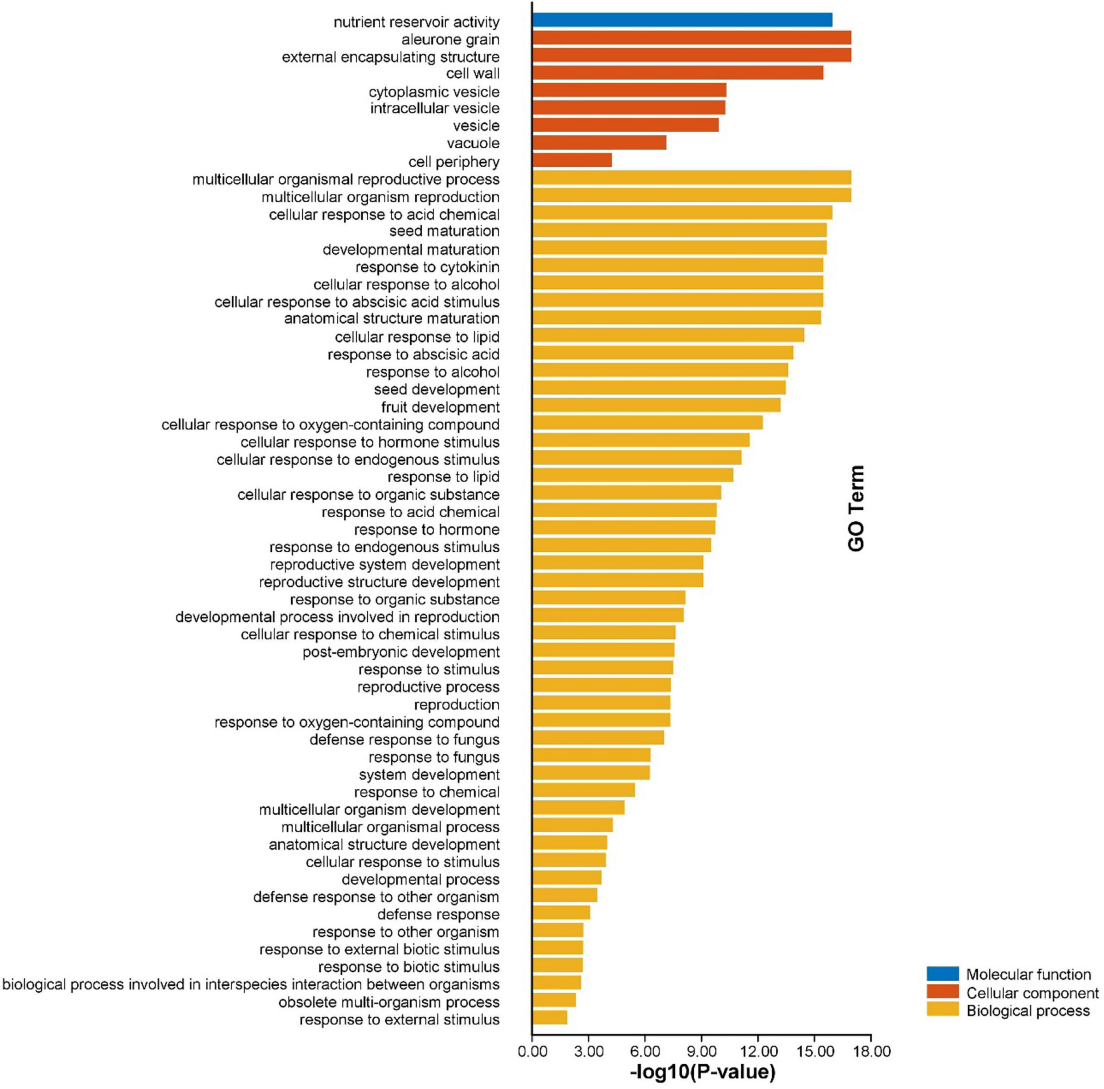
Functional annotation (GO) analysis of *AhGLPs*. The gene ontology enrichment analysis showed that *AhGLPs* are involved in all categories, including molecular function (MF), cellular component (CC), and biological processes (BP). *AhGLPs* are involved in a number of important biological processes, including growth and development related, biotic and abiotic stress response, and various stimulus responses.

Gene ontology (GO) enrichment analysis of *AhGLPs* was performed to view their possible roles in biological processes (BP), molecular functions (MF), and cellular components (CC). GO enrichment results provided highly enriched terms related to BP, MF, and CC (**Figure 10**). *AhGLPs* were involved in several biological processes, including reproductive processes (GO:0048609 and GO:0032504), seed and fruit development (GO:0010431, GO:0048316, and GO:0010154), defense responses against various biotic and abiotic agents (GO:0050832, GO:0009620, GO:0098542), response to various phytohormones (GO:0009725, GO:0009735), interspecies interaction (GO:0044419), response to different chemicals (GO:0042221), responses to other organisms (GO:0098542), response to different endogenous and exogenous stimuli (GO:0043207, GO:0009607, GO:0050896), and postembryonic development (GO:0009791) etc. In the cellular component category (CC), *AhGLPs* are part of the cell wall (GO:0005618), cytoplasmic vesicle (GO:0031410), vesicle (GO:0031982), vacuole (GO:0005773), etc. For the molecular function category (MF), *AhGLPs* are involved in nutrient reservoir activity (GO:0045735) (the main molecular function of GLPs indeed). The detailed information about MF, BP, and CC categories, their associated GO IDs, and *AhGLP* members involved in these categories are given in **Figure 11** and **Supplementary File 4**. Collectively it is evident from functional annotation analysis that *AhGLPs* play key roles in several biological, cellular, and molecular functions.

### 3.9 Expression profiling in different tissues and under different abiotic stresses

**Figure 12:**
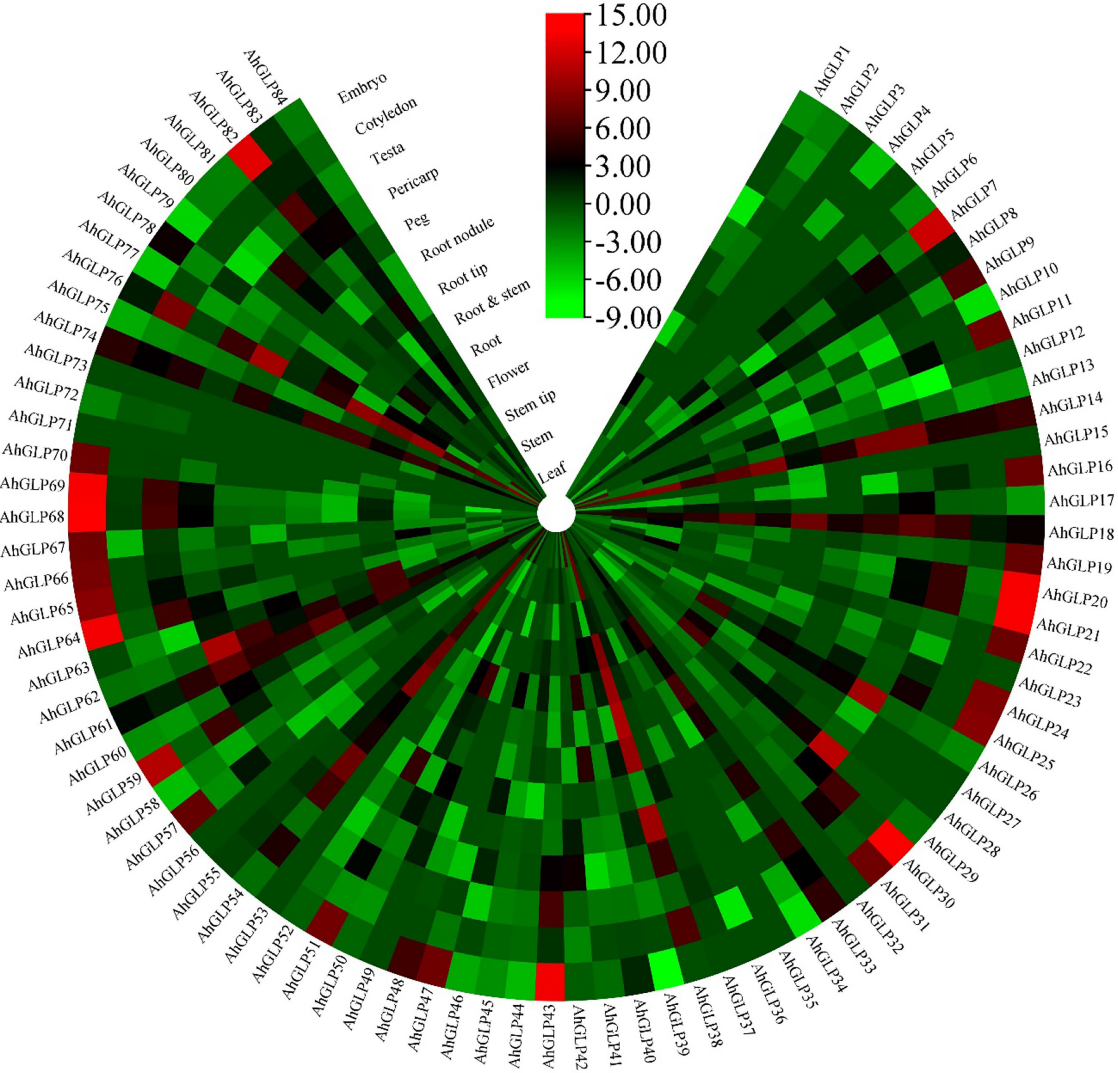
Transcriptome expression of *AhGLPs* in different tissues. *AhGLPs* possessed varying expression matrices in different tissues. Some genes are very lowly expressed in all tissues, while others are specifically expressed in leaf, pericarp, and embryo.

**Figure 13:**
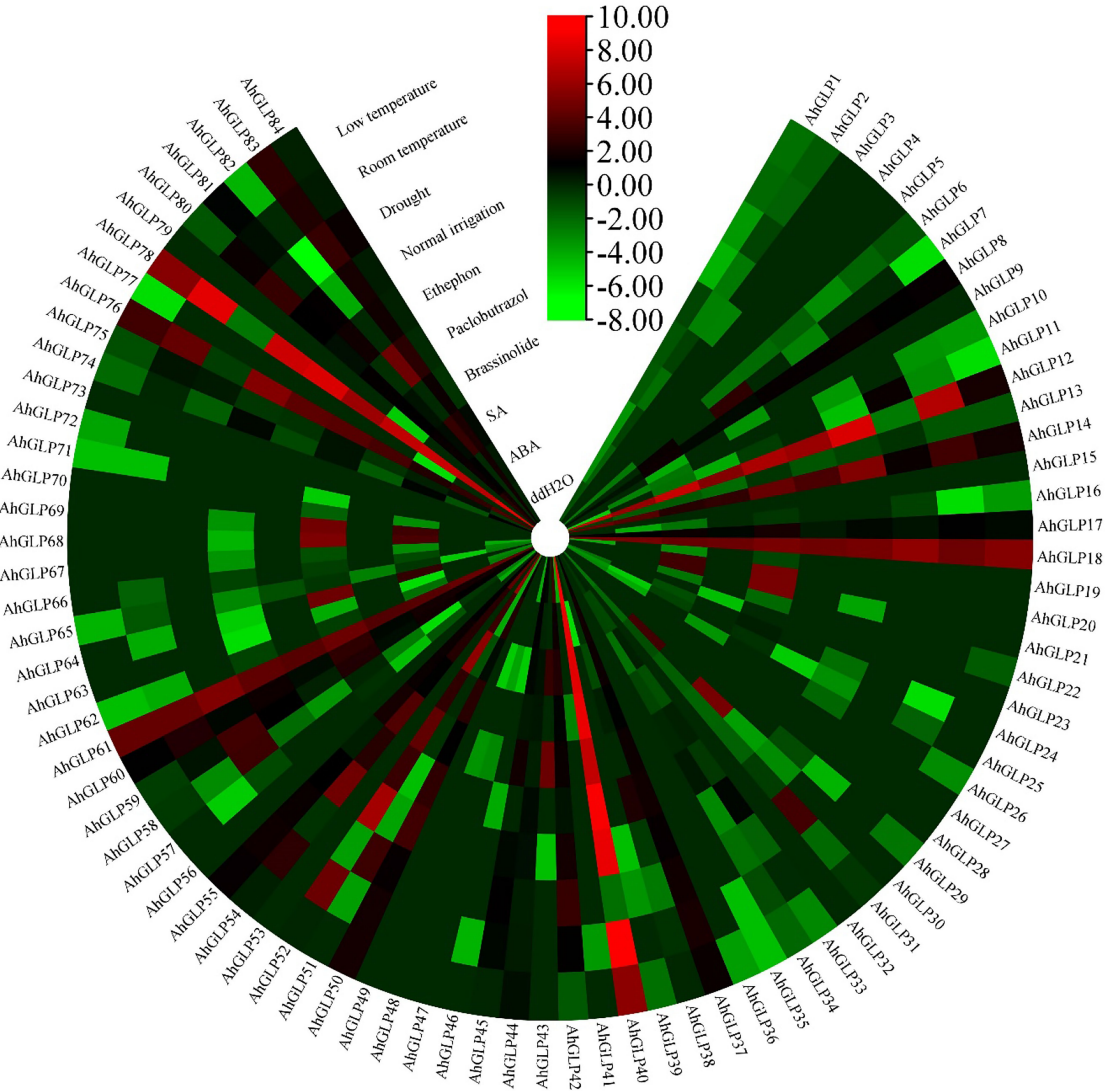
Transcriptome expression of *AhGLPs* under different hormones and stress conditions. Most of AhGLPs are not responsive to different stress treatments, while few genes *AhGLP12*, *AhGLP18*, *AhGLP40*, *AhGLP61*, and *AhGLP78* showed good responses to all stress/normal environments.

Transcriptome expression data was used to view the expression levels of 84 *AhGLPs* in different tissues and under different abiotic stresses. *AhGLPs* possessed diverse expressions in different peanut tissues. Almost 25 genes showed expression responses in the embryo, including *AhGLP7*, *AhGLP9*, *AhGLP11*, *AhGLP16*, *AhGLP19*, *AhGLP24*, *AhGLP47*, *AhGLP57*, *AhGLP66*, *AhGLP67*, *AhGLP68, AhGLP69*, *AhGLP70*, *AhGLP80* etc. Genes, including *AhGLP14*, *AhGLP26*, *AhGLP29*, and *AhGLP62*, were abundantly expressed in the pericarp. *AhGLP3*, *AhGLP5*, *AhGLP32*, *AhGLP53*, *AhGL71*, and *AhGLP73* were uniquely down regulated in all tissues (**Figure 12**). Transcriptome expression data against different phytohormones including ABA, SA, Brassinolide, Paclobutrazol, and Ethephon treatment, water stress (drought and normal irrigation), temperature stress (low 4°C and room temperature 28°C) were also accessed to determine their stress responses. *AhGLP12*, *AhGLP14*, *AhGLP18*, *AhGLP40*, *AhGLP52*, *AhGLP61*, and *AhGLP78* were upregulated under normal and hormonal treatment (**Figure 13**). *AhGLP20*, *AhGLP21*, *AhGLP30*, *AhGLP43*, *AhGLP60*, *AhGLP64*, *AhGLP68*, *AhGLP69*, and *AhGLP82* were upregulated under paclobutrazol treatment as compared to control and other hormones. The expression responses of all genes to other hormones were similar to control. The expression responses of *AhGLPs* under different water treatments (drought and normal irrigation) were comparable to hormones. Some genes, including *AhGLP12*, *AhGLP14*, *AhGLP24*, *AhGLP52*, *AhGLP54*, and *AhGLP74*, were decreased under drought stress as compared to normal irrigation. But *AhGLP31*, *AhGLP42*, *AhGLP50*, *AhGLP58*, and *AhGLP69* showed little increase under drought stress as compared to normal irrigation. A similar pattern was also observed under different temperature (low and normal) treatments. A few genes (*AhGLP12*, *AhGLP14*, *AhGLP52*, and *AhGLP54*) also showed decreased expression under low temperatures. The Fragments Per Kilobase Million (FPKM) values of transcriptome expression of *AhGLPs* in different tissues and under different stress conditions are given in **Supplementary Tables 7, 8**.

### 3.10 Real-time expression of *AhGLPs* under cold and ABA treatments

**Figure 14:**
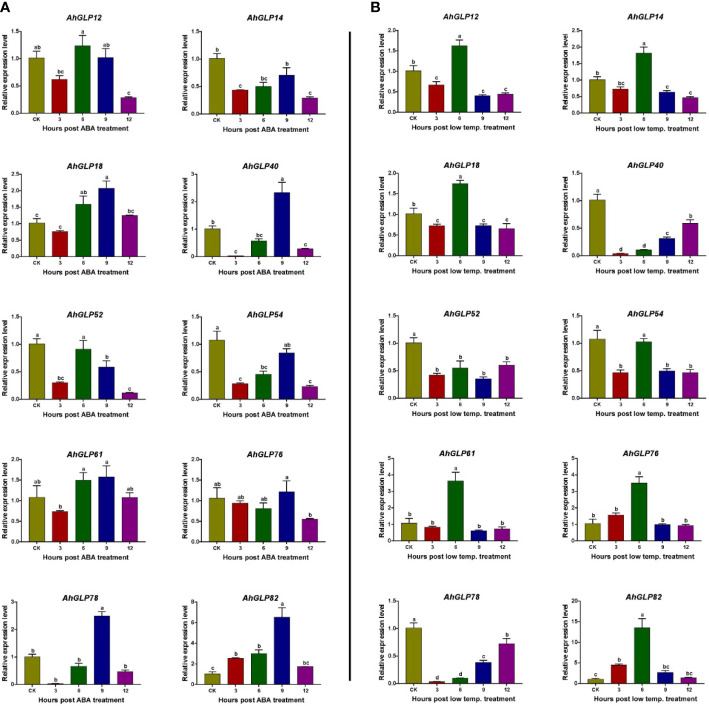
qRT-PCR-based expression profiling of 10 selected genes under **(A)** abscisic acid and **(B)** low-temperature stress. CK represents the control samples, while 3, 6, 9, and 12 represent the hours after stress (ABA/low temperature) treatment. Data were analyzed by 2^-ΔΔCT^ method and statistical significance was determined by ANOVA. a, b, and c represents the significance levels among expression at different time points.

For the validation of expression matrices, ten *AhGLP* genes with high transcriptome expression in response to ABA and low temperature (4 °C) were selected (**Figure 13**), and their expression was monitored with the help of quantitative real-time PCR. Most of the selected genes showed increased expression in response to ABA treatment except a few genes showed lower expression. Genes *AhGLP14*, *AhGLP52* and *AhGLP54* were downregulated in response to ABA treatment at all time points as compared to control. The expression of *AhGLP14* and *AhGLP54* was less downregulated at 9h, while the expression of *AhGLP52* was less downregulated at 6h post-ABA treatment (**Figure 14** **A**). Under low-temperature stress, most of the genes were upregulated, except *AhGLP40*, *AhGLP52*, and *AhGLP78*, which were downregulated (**Figure 14** **B**). *AhGLP40* showed a gradual increase in expression level but still its expression was lower under low temperature than control. The expression level of *AhGLP54* at 9h was comparable to control, but it was downregulated at all other time points. In most cases, expression was highest at 6h after low-temperature treatment. Although there were some expression variations at different time points, overall, the qRT-PCR-based expression is in accordance with transcriptome expression. These results represent the reliability of the transcriptome expression data.

## 4 Discussion

Peanut (*Arachis hypogaea* L.) is an important legume crop providing edible oil, proteins, food, and feed to humans and livestock. It is a staple grain in many Asian and African countries and constitutes a major part of their every meal. Like other crops, peanut has to face various bacterial, viral, and fungal pathogens and harsh environmental conditions (Zhang et al., 2017; Khan et al., 2020). Crops have evolved many defense mechanisms to combat unfavorable conditions. Genome duplications, gene family evolution, and seed dispersal mechanisms are among the strategies that plants adopt to survive. Different gene families have important roles in the plant kingdom. Thanks to the advances of functional genomics and high throughput sequencing technologies that have made it easy to identify the gene families and explore their roles in different metabolic and defense pathways. *GLP* family is among plant gene families playing key roles in plant defense and growth regulation (Wang et al., 2013). Previously *GLP* gene family has been studied at a genome-wide scale in some important crop species. Different numbers of *GLP* family members are found in different plants, mainly depending upon their ploidy level and genome size. To date, 32 GLP genes in *Arabidopsis* and 43 in rice (Li et al., 2016), 48 in barley (Zimmermann et al., 2006), 69 in soybean (Wang et al., 2014), and 258 in wheat (Yuan et al., 2021) have been reported. Based on the *Arabidopsis* GLPs and germin/Cupin domain (PF00190) search, we identified 37, 32, and 84 GLPs in *A. duranensis*, *A. ipaensis*, and *A. hypogaea*, respectively. The consensus sequences of cupin motifs in peant GLPs are given in **Supplementary File 1**. The number of genes belonging to a group/family in a species depends on genome size and complexity. The number of GLP homologs in *Arabidopsis* are lowest among all studied species, which is mainly due to smaller genome size and a smaller number of chromosomes. Previous studies reported 32 GLPs in *Arabidopsis* (Li et al., 2016), but we identified one more GLP member in *Arabidopsis* with careful analysis. Number of GLP homologs in peanut species show similar pattern as of other plant species. GLPs in diploid peanut species (*A. duranensis* and *A. ipaensis*) are very close to *Arabidopsis*. While the complex genome of *A. hypogaea* possessed a higher number of GLP homologs, as its genome is composed of two subgenomes that have undergone natural duplication, resulting in an allotetraploid species.

The large genome size and tetraploid nature of *A. hypogaea* genome have resulted in more *GLPs* than its diploid progenitors. Structural diversity of gene families has resulted due to the evolutionary process, and the number of exons/introns plays a key role in the expression of a gene (Hu et al., 2010; Guo and Qiu, 2013). Generally, the germin-like protein family is characterized by two exons (Li et al., 2016), but exon/intron number variations are very common. Many GLPs of wild peanut species were composed of two exons and a single intron (**Supplementary Figures 3, 4**). The maximum number of exons exceeded seven for *A. duranensis* and 15 for *A. ipaensis*. In contrast, most *AhGLPs* were composed of a single exon (**Figure 2**), and the maximum number of exons is 10 (**Table 1**). The presence of a single exon in most of *AhGLPs* might be due to intron deletion during the evolutionary process. Genome size variations and gene duplication events are key elements of genetic diversity. As genomic duplication is a key factor underlying gene families’ expression, diversification, and neofunctionalization, we also analyzed the duplication events in *AhGLPs*. Duplication analysis revealed 38 duplicated gene pairs in *AhGLPs* with varying divergence times (**Table 2**). Mostly segmental duplication events were found in the evolution of peanut germin-like proteins, and this type of duplication has been reported previously in rice and *Arabidopsis* (Li et al., 2016). The phylogenetic analysis previously divided the GLP family into six groups (Yuan et al., 2021); six groups were also found in peanut GLPs. In the phylogenetic classification, all GLPs of *Arabidopsis* were restricted to four groups, as described in previous studies (Li et al., 2016; Yuan et al., 2021). But the germin-like proteins of wild and cultivated peanut species were distributed among all six groups depicting a close relationship among peanut species (**Figure 4**).

*Cis*-elements of a genes’ promoter are significant as they determine the expression pattern, regulatory roles, and stress responses (Maruyama-Nakashita et al., 2005; Osakabe et al., 2014). We identified four types of *cis*-elements in *AhGLPs* promoters: light-responsive, hormones-responsive, growth and regulation-related and stress-related elements, and seed-specific expression-related elements (RY-repeat) were also found in some of *AhGLPs* (**Figure 6**). Micro-RNAs are critical regulatory elements and play important roles against different stresses (Su et al., 2021). For the past few years, miRNAs have been a hot research topic (Xie and Zhang, 2015; Ding et al., 2017; Chen et al., 2019). The present study predicted the putative miRNAs targeting *AhGLPs* from the published miRNAs database (Dai et al., 2018). We found seven miRNAs belonging to six families, targeting 25 *AhGLPs* (**Supplementary Table 6** and **Figure 7**). Among these miRNAs, miRNA408 have been reported to be involved in responses to water deficit conditions, whereas it is upregulated in *M. truncatula* (Trindade et al., 2010) and downregulated in pea (Jovanović et al., 2014). It is also involved in drought tolerance in chickpea (Hajyzadeh et al., 2015). miR394 has been reported to improve drought and salt tolerance in *Arabidopsis* (Song et al., 2013), while miR167 has been reported to regulate the auxin response genes under blue light in *Arabidopsis* (Pashkovskiy et al., 2016). These reports suggest that predicted miRNAs targeting the *AhGLPs* might play key roles in response to different abiotic stresses.

Transcriptome-based expression profiling of *AhGLPs* revealed their expression under different abiotic stresses and in different tissues. A fraction of *AhGLPs* showed embryo-specific expression (**Figure 12**), while a few gens showed pericarp-specific expression. Interestingly pericarp specific genes (*AhGLP26*, *AhGLP29*, and *AhGLP62*) possessed the longest genomic sequences and longest introns among all *AhGLPs* (**Figure 2** and **Table 1**), indicating some special role of long introns in pericarp-specific expression. Expression profiling under different stresses revealed their responses to drought and low temperature. These results are supported by previous studies on *GLP* genes (Lee and Lee, 2003; Komatsu et al., 2010). Hormones regulate a genes’ biochemical and physiological reactions through different signal transduction pathways (Fatima et al., 2021; Zaynab et al., 2021; Raza et al., 2022b). Drought is a major limiting factor for crop yields. And drought-smart breeding is a need of the day (Raza et al., 2022a). Transcriptome expression data revealed that some GLPs genes could be used for drought-smart breeding also. *AhGLPs* showed varying responses to different hormones treatment. Most of the genes were not affected by hormone treatment, some genes were downregulated, and a few were upregulated (**Figure 12**). Similar findings have also been reported for ABA, salt and drought stress (Wang et al., 2013; Liao et al., 2021). In response to different water and temperature treatments, a similar expression pattern was observed as in the case of hormones. *AhGLP12*, *AhGLP18*, *AhGLP40*, *AhGLP61*, and *AhGLP78* showed higher expression under all hormones, water, and temperature treatments. Similar findings were also found by Huang et al. (2020). Similarly, drought is also a limiting factor and makes developing peanut seeds vulnerable to many pathogens. They performed RNA sequencing of peanut cultivar “Hua Yu 39” in response to Brassinolide treatment under drought and normal irrigation conditions (Huang et al., 2020). Their findings also support our transcriptome expression results. The qRT-PCR-based expression profiling of selected genes provided the validity of transcriptome expression. It provided some key genes responsive to ABA treatment, including, *AhGLP18*, *AhGLP78* and *AhGLP82*. Similarly, real-time expression profiling of selected genes under low temperature provided that *AhGLP12*, *AhGLP18*, *AhGLP61*, *AhGLP76*, and *AhGLP82* are key for low temperature responses. Overall, this study provides a deep insight into the molecular evolution and functioning of *AhGLPs* in cultivated peanut. And this study will be a base for further work on peanut germin-like proteins. Along with genomics, gene editing tools could be applied for orphan crop improvement, including cultivated peanut (Yaqoob et al., 2023).

## 5 Conclusion

This study identified 84 *AhGLPs* genes in the genome of cultivated peanut through a comprehensive genome-wide analysis. In the diploid progenitors of *A. hypogaea* (*A. duranensis* and *A. ipaensis*), 37 and 32 *GLPs* genes were identified, respectively. Phylogenetic and syntenic relations and gene structure analysis provided more profound insight into the evolutionary history of *AhGLPs*. Predicted miRNAs have shown their defensive roles against abiotic stress tolerance. Hence, they provide a future research dimension for stress-smart breeding. The transcriptome analysis showed that some genes responded significantly to hormones and abiotic stress stimuli. Some genes showed specific expression in pericarp and embryo, such as *AhGLP7*, *AhGLP20*, *AhGLP21*, *AhGLP30*, *AhGLP43*, *AhGLP64*, *AhGLP68*, *AhGLP69*, and *AhGLP82*. These genes and their promoters are valuable future candidates to express in embryo-specific manner or drive a gene in embryo-specific manner. Thus, additional investigations are required to confirm the purposeful role of *GLPs* in peanut growth, development, and response to numerous environmental stresses.

## Data availability statement

The datasets presented in this study can be found in online repositories. The names of the repository/repositories and accession number(s) can be found below: https://www.ncbi.nlm.nih.gov/bioproject/PRJNA480120.

## Author contributions

WZ and HC conceived the idea and designed the study. QY, YS, YZ, HF, and SW analyzed the data and wrote the manuscript. CZ, TC, QY, KC, AR, and LW helped in literature search, and provided technical guidance. WZ, HC, and YZ supervised the work and edited the final version. QY and YS equally contributed to the manuscript. All authors have read and approved the final version of the manuscript.
