# Supplementary material for: Genome-wide identification of germin-like proteins in peanut (Arachis hypogea L.) and expression analysis under different abiotic stresses
Source: Front Plant Sci. 2023 Jan 23;13:1044144. doi: 10.3389/fpls.2022.1044144 (PMC9901545; doi:10.3389/fpls.2022.1044144)
Supplement: Supplementary file 1 [file DataSheet_1.zip › Image 1.pdf]

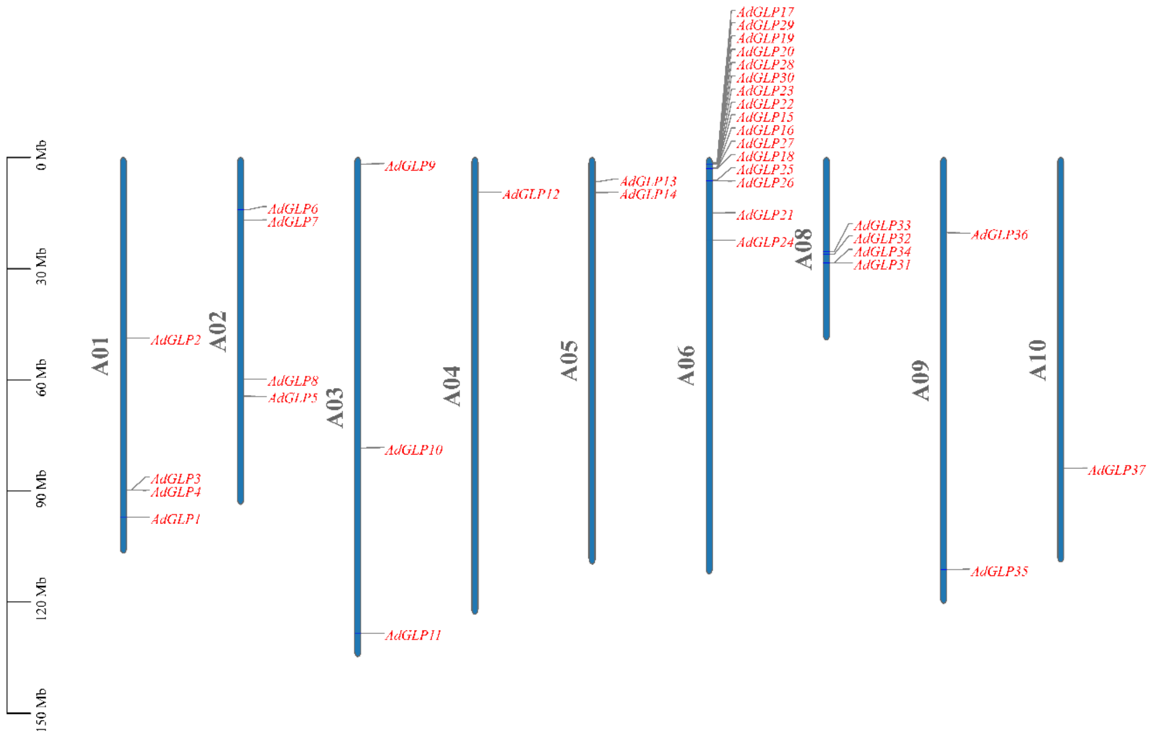

Supplementary Figure 1. Chromosomal distribution of *Arachis duranensis* Germin-like protein (*AdGLPs*) genes. Gernim-like proteins are unevenly distributed in the genome of *A. duranensis*. ChrA06 possessed the highest number of GLPs (16 GLPs).

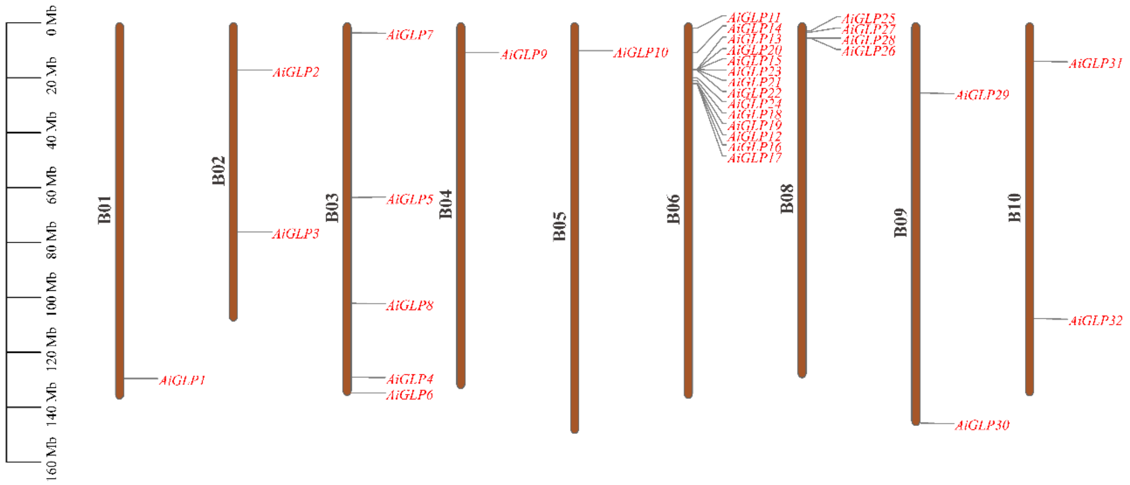

Supplementary Figure 2. Chromosomal distribution of *Arachis ipaensis* Germin-like protein (*AiGLPs*) genes. Gernim-like proteins are unevenly distributed in the genome of *A. ipaensis*. ChrA03 possessed the highest number of GLPs (14 GLPs).

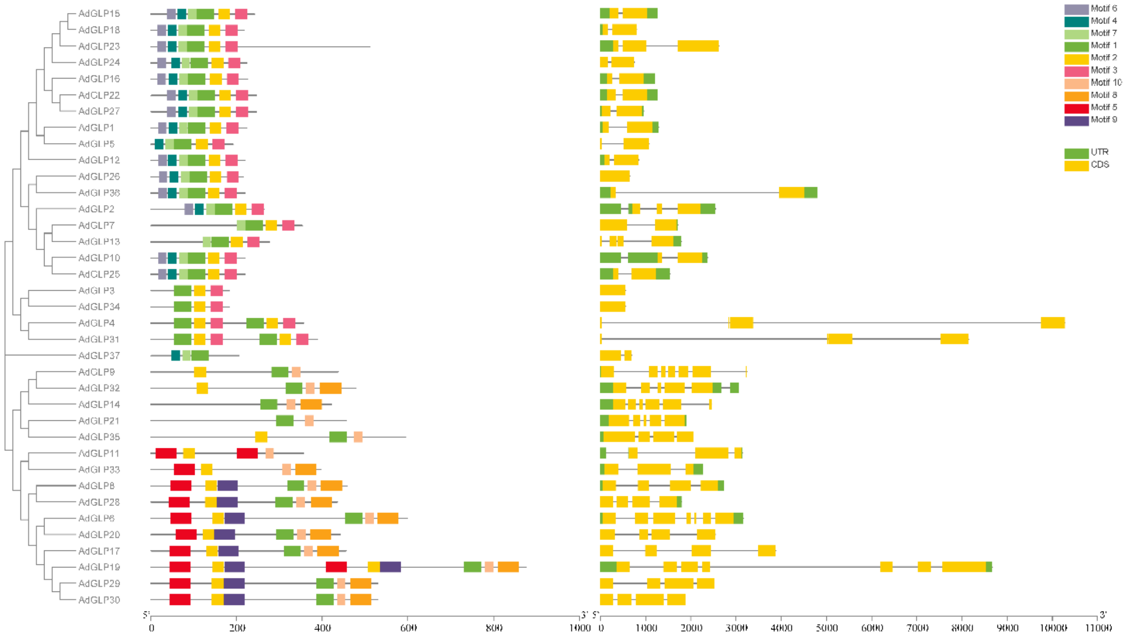

Supplementary Figure 3. Conserved motifs and gene structure (exons-introns distribution) analysis of *Arachis duranensis* Germin-like protein (*AdGLPs*) genes.

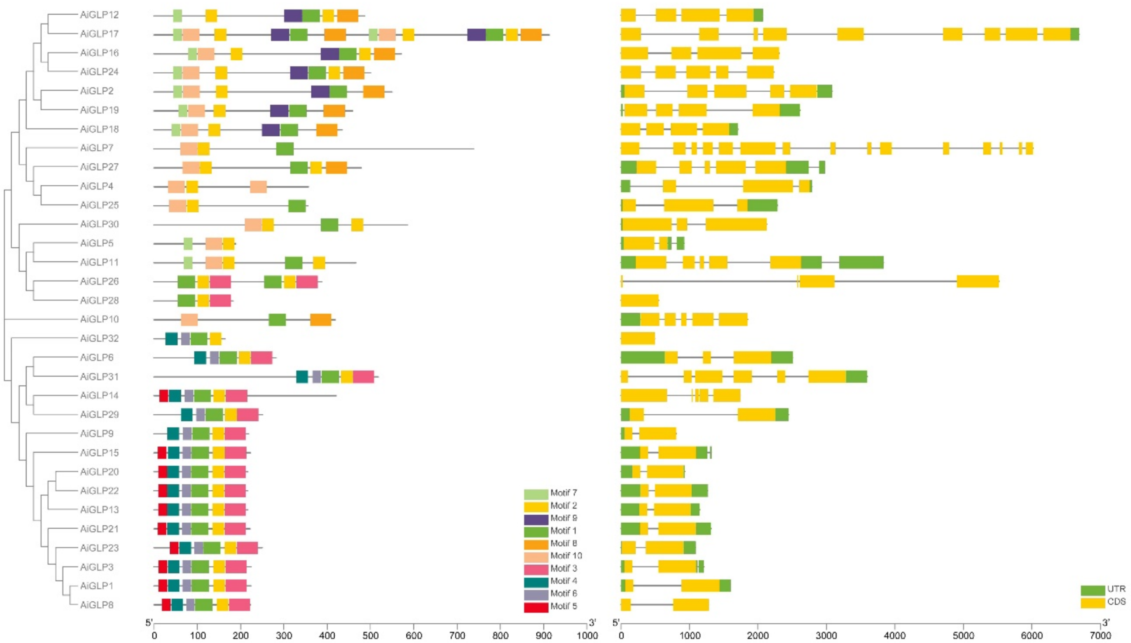

Supplementary Figure 4. Conserved motifs and gene structure (exons-introns distribution) analysis of *Arachis ipaensis* Germin-like protein (*AiGLPs*) genes.

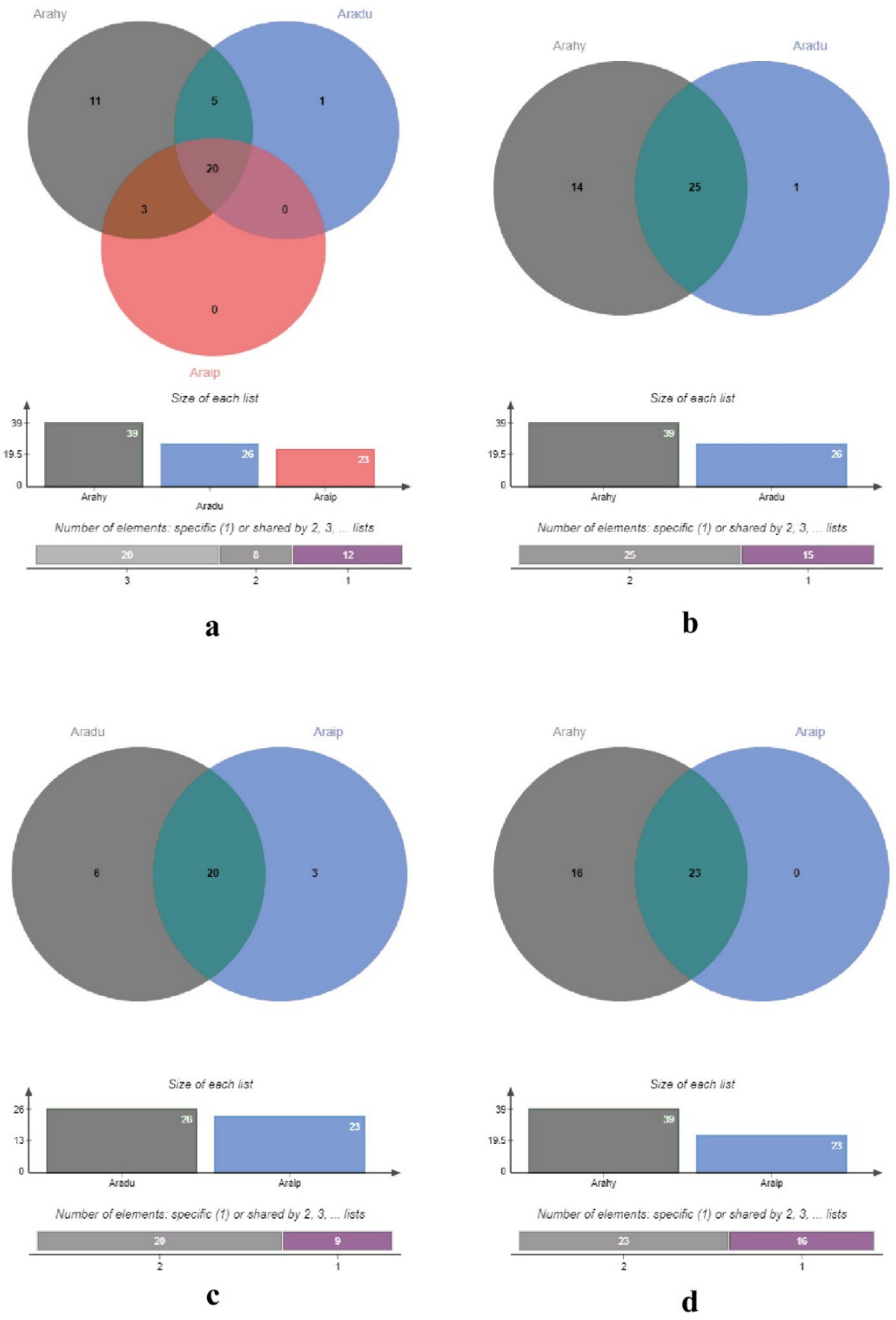

Supplementary Figure 5. Orthologous genes' clusters among diploid and tetraploid species of peanut. (a) orthologous gene clusters among *A. hypogea*, *A. duranensis*, and *A. ipaensis* (b) orthologous gene clusters among *A. hypogea* and *A. duranensis* (c) orthologous gene clusters among *A. duranensis* and *A. ipaensis* (d) orthologous gene clusters among *A. hypogaea* and *A. ipaensis*.
