# Supplementary material for: Genome-wide identification of germin-like proteins in peanut (Arachis hypogea L.) and expression analysis under different abiotic stresses
Source: Front Plant Sci. 2023 Jan 23;13:1044144. doi: 10.3389/fpls.2022.1044144 (PMC9901545; doi:10.3389/fpls.2022.1044144)
Supplement: Supplementary file 1 [file DataSheet_1.zip › Table 5.docx]

Supplementary Table 5. 10 identified motifs of *AiGLPs*

| Sr. no | Motif sequence | **E-value** | **Sites** | **Width** |
| --- | --- | --- | --- | --- |
| 1 | **NTLGISLARIDFGPKGLNPPHTHPRATEILIVIEGTLLVGF** | 4.4e-423 | 29 | 41 |
| 2 | **NRLFTKVLNKGDVFVFPIGLIHFQFNVG** | 1.30E-288 | 28 | 28 |
| 3 | **YGNAVAISGLSSQNPGVITIANAVFGSTPPISPEVLTKAFQVDKKVINYL** | 1.2e-317 | 14 | 50 |
| 4 | **AVNDTKSAVFVNGKFCKDPMLVVAEDFF** | 5.40E-148 | 14 | 28 |
| 5 | **LALASSFASAYDPSPLQDFCV** | 5.20E-100 | 10 | 21 |
| 6 | **NVVNKLGSNVTPVSVNELPGL** | 5.00E-97 | 14 | 21 |
| 7 | **RIESEGGYIETWNPNNQEFEC** | 3.10E-62 | 9 | 21 |
| 8 | **AFKTDSRPSIANLAGENSFIDNLPEEVVANSYGLPREQARQLKNNNPFKF** | 4.50E-81 | 6 | 50 |
| 9 | **NGIEETICTATVKMNIGKSTSADIYNPQAGSVRTVNELDLPIL** | 3.40E-79 | 7 | 43 |
| 10 | **GATLSRCTLRRNALRRPYYSNAPQQIFIQQGSGYFGLIFP** | 7.90E-78 | 14 | 40 |
