# Supplementary material for: Genome-wide identification of germin-like proteins in peanut (Arachis hypogea L.) and expression analysis under different abiotic stresses
Source: Front Plant Sci. 2023 Jan 23;13:1044144. doi: 10.3389/fpls.2022.1044144 (PMC9901545; doi:10.3389/fpls.2022.1044144)
Supplement: Supplementary file 1 [file DataSheet_1.zip › Table 4.docx]

Supplementary Table 4. 10 identified motifs of *AdGLPs*

| Sr. no | Motif sequence | **E-value** | **Sites** | **Width** |
| --- | --- | --- | --- | --- |
| 1 | **LNTLGVSLARIDYAPGGLNPPHTHPRATEIVFVLEGQLDVG** | 8.8e-590 | 35 | 41 |
| 2 | **NRLFTKVLNKGDVFVFPIGLIHFQFNVG** | 2.7e-375 | 33 | 28 |
| 3 | **SQNPGVITIANAVFGSTPPISPEVLTKAF** | 4.10E-250 | 19 | 29 |
| 4 | **FVNGKFCKDPKLVVAEDFFKH** | 3.10E-131 | 15 | 21 |
| 5 | **RIESEGGYIETWNPNNQEFECAGVALSRLVLRRNALRRPFYSNAPQEIFI** | 1.70E-148 | 8 | 50 |
| 6 | **SFASAYDPSPLQDFCVALPDG** | 2.70E-122 | 14 | 21 |
| 7 | **GNVDNKLGSNVTPVSVNELPG** | 1.20E-119 | 18 | 21 |
| 8 | **YVAFKTDSRPSIANLAGENSIIDNLPEEVVANSYGLPREQARQLKNNNPF** | 2.90E-97 | 7 | 50 |
| 9 | **DTDVVAISLTDTNNNDNQLDQFPRRFNLAGNQEQEFLRYQQQSRQSRRR** | 1.40E-90 | 8 | 49 |
| 10 | **RVYDEELQEGHVLVVPQNFAV** | 4.50E-71 | 17 | 21 |
