# Supplementary material for: Genome-wide identification of germin-like proteins in peanut (Arachis hypogea L.) and expression analysis under different abiotic stresses
Source: Front Plant Sci. 2023 Jan 23;13:1044144. doi: 10.3389/fpls.2022.1044144 (PMC9901545; doi:10.3389/fpls.2022.1044144)
Supplement: Supplementary file 1 [file DataSheet_1.zip › Table 3.docx]

Supplementary Table 3. 10 identified motifs of *AdGLPs*

| Sr. no | 10 identified motifs of *AhGLPs* | **E-value** | **Sites** | **Width** |
| --- | --- | --- | --- | --- |
| 1 | **GGLNPPHTHPRATEIVFVLEGQL** | 1.9e-845 | 79 | 23 |
| 2 | **KVLNKGDVFVFPKGLIHFQLN** | 8.1e-772 | 82 | 21 |
| 3 | **VTPVSVNELPGLNTLGISLAR** | 4.2e-429 | 53 | 21 |
| 4 | **GYGNALAIAALSSQNPGVITIANAVFGST** | 3.3e-532 | 49 | 29 |
| 5 | **ESEGGYIETWNPNNQEFECAGVALSRLVLRRNALRRPFYSNAPQEIFIQQ** | 5.8e-486 | 17 | 50 |
| 6 | **FKTDSRPSIANLAGENSIIDNLPEEVVANSYRLPREQARQLKNNNPFKFF** | 7.1e-435 | 17 | 50 |
| 7 | **VNGKFCKDPKVVVAEDFFKHV** | 1.0e-350 | 42 | 21 |
| 8 | **HVQVVDSNGNRVYDEELQEGHVLVVPQNFA** | 2.1e-338 | 26 | 30 |
| 9 | **DHDTDVVAVSLTDTNNNDNQLDQFPRRFNLAGN** | 1.80E-294 | 19 | 33 |
| 10 | **SFASAYDPSPLQDFCVALPDG** | 1.30E-282 | 42 | 21 |
