# Supplementary material for: Genome-wide identification of germin-like proteins in peanut (Arachis hypogea L.) and expression analysis under different abiotic stresses
Source: Front Plant Sci. 2023 Jan 23;13:1044144. doi: 10.3389/fpls.2022.1044144 (PMC9901545; doi:10.3389/fpls.2022.1044144)
Supplement: Supplementary file 1 [file DataSheet_1.zip › Table 2.docx]

Supplementary Table 2. Germin-like protein members in *Arachis ipaensis* and their physiochemical properties

| Gene | Transcript ID | Chr | Gene (bp) | CDS (bp) | Protein (aa) | Exons | MW (KDa) | pI | Subcellular localization |
| --- | --- | --- | --- | --- | --- | --- | --- | --- | --- |
| *AiGLP1* | Araip.K4ZG6 | B01 | 1601 | 675 | 224 | 1 | 24.29472 | 6.89 | Extracellular |
| *AiGLP2* | Araip.FAG7U | B02 | 3083 | 1653 | 550 | 5 | 62.94363 | 6.1 | Extracellular/Nuclear |
| *AiGLP3* | Araip.V8RD2 | B02 | 1211 | 675 | 224 | 2 | 24.19668 | 6.28 | Extracellular |
| *AiGLP4* | Araip.06WGU | B03 | 2790 | 1074 | 357 | 3 | 38.46907 | 5.45 | Cytoplasmic/Chloroplast |
| *AiGLP5* | Araip.30GQD | B03 | 927 | 570 | 189 | 2 | 21.98492 | 5.75 | Extracellular/Nuclear |
| *AiGLP6* | Araip.FD663 | B03 | 2510 | 849 | 282 | 3 | 29.71157 | 9.4 | PlasmaMembrane |
| *AiGLP7* | Araip.I9427 | B03 | 6018 | 2217 | 738 | 15 | 82.6075 | 5.72 | Cytoplasmic/Nuclear |
| *AiGLP8* | Araip.UNX5Q | B03 | 1283 | 672 | 223 | 2 | 23.9854 | 5.91 | Extracellular/PlasmaMembrane |
| *AiGLP9* | Araip.7L3C8 | B04 | 810 | 660 | 219 | 2 | 23.31455 | 6.13 | Extracellular |
| *AiGLP10* | Araip.MGW36 | B05 | 1852 | 1260 | 419 | 5 | 46.55011 | 8.17 | Extracellular |
| *AiGLP11* | Araip.16S9Q | B06 | 3835 | 1401 | 466 | 5 | 53.01923 | 6.18 | Cytoplasmic/Nuclear |
| *AiGLP12* | Araip.5JB56 | B06 | 2074 | 1464 | 487 | 4 | 55.31891 | 5.23 | Nuclear |
| *AiGLP13* | Araip.99Z22 | B06 | 1151 | 654 | 217 | 2 | 23.35285 | 6.4 | Extracellular |
| *AiGLP14* | Araip.ADC8R | B06 | 1744 | 1266 | 421 | 5 | 46.33296 | 6.32 | PlasmaMembrane |
| *AiGLP15* | Araip.B0Q1D | B06 | 1324 | 672 | 223 | 2 | 24.06368 | 6.89 | Extracellular |
| *AiGLP16* | Araip.D61U9 | B06 | 2312 | 1716 | 571 | 4 | 65.21118 | 5.57 | Extracellular/PlasmaMembrane |
| *AiGLP17* | Araip.IGC50 | B06 | 6689 | 2742 | 913 | 9 | 103.51198 | 6.04 | Extracellular/Cytoplasmic/Nuclear |
| *AiGLP18* | Araip.LRG7E | B06 | 1709 | 1308 | 435 | 4 | 47.84648 | 5.33 | Cytoplasmic |
| *AiGLP19* | Araip.SK1EN | B06 | 2617 | 1380 | 459 | 4 | 50.48476 | 5.8 | Chloroplast |
| *AiGLP20* | Araip.TS8PF | B06 | 934 | 654 | 217 | 2 | 23.33887 | 6.9 | Extracellular |
| *AiGLP21* | Araip.UJ97I | B06 | 1318 | 669 | 222 | 2 | 23.93555 | 6.89 | Extracellular |
| *AiGLP22* | Araip.UR5L6 | B06 | 1269 | 654 | 217 | 2 | 23.33887 | 6.9 | Extracellular |
| *AiGLP23* | Araip.WB5CY | B06 | 1091 | 753 | 250 | 2 | 27.4838 | 9.54 | Extracellular/PlasmaMembrane |
| *AiGLP24* | Araip.WQE9Q | B06 | 2232 | 1503 | 500 | 5 | 56.80682 | 5.95 | Nuclear |
| *AiGLP25* | Araip.IF9S9 | B08 | 2286 | 1071 | 356 | 3 | 38.24582 | 5.78 | Chloroplast |
| *AiGLP26* | Araip.Q61XZ | B08 | 5520 | 1167 | 388 | 4 | 42.17141 | 5.59 | PlasmaMembrane |
| *AiGLP27* | Araip.QU0FD | B08 | 2979 | 1440 | 479 | 5 | 53.83764 | 5.61 | Cytoplasmic |
| *AiGLP28* | Araip.RTN15 | B08 | 552 | 552 | 183 | 1 | 19.74939 | 5.16 | Extracellular |
| *AiGLP29* | Araip.J6PP8 | B09 | 2448 | 756 | 251 | 2 | 26.76192 | 8.49 | PlasmaMembrane |
| *AiGLP30* | Araip.T82B5 | B09 | 2129 | 1761 | 586 | 3 | 66.84345 | 7.67 | Nuclear |
| *AiGLP31* | Araip.F3HB9 | B10 | 3596 | 1557 | 518 | 6 | 55.77729 | 8.38 | PlasmaMembrane |
| *AiGLP32* | Araip.QIV9F | B10 | 495 | 495 | 164 | 1 | 18.06474 | 6.49 | Extracellular/PlasmaMembrane |

Note. Chr=Chromosome, MW=molecular weight, pI= theoretical isoelectric point
