# Supplementary material for: Genome-wide identification of germin-like proteins in peanut (Arachis hypogea L.) and expression analysis under different abiotic stresses
Source: Front Plant Sci. 2023 Jan 23;13:1044144. doi: 10.3389/fpls.2022.1044144 (PMC9901545; doi:10.3389/fpls.2022.1044144)
Supplement: Supplementary file 1 [file DataSheet_1.zip › Table 1.docx]

Supplementary Table 1. Germin-like protein members in *Arachis duranensis* and their physiochemical properties

| Gene | Transcript ID | Chr | Gene (bp) | CDS (bp) | Protein (aa) | Exons | MW (KDa) | pI | Subcellular localization |
| --- | --- | --- | --- | --- | --- | --- | --- | --- | --- |
| *AdGLP1* | Aradu.7R15V | A01 | 1294 | 675 | 224 | 2 | 24.30472 | 6.41 | Extracellular |
| *AdGLP2* | Aradu.90S6X | A01 | 2548 | 795 | 264 | 3 | 28.01846 | 8.89 | PlasmaMembrane |
| *AdGLP3* | Aradu.HF645 | A01 | 552 | 552 | 183 | 1 | 19.72133 | 5.16 | Extracellular |
| *AdGLP4* | Aradu.QK4SE | A01 | 10278 | 1074 | 357 | 4 | 39.14283 | 6.24 | PlasmaMembrane |
| *AdGLP5* | Aradu.3SF2D | A02 | 1070 | 579 | 192 | 2 | 20.95991 | 6.18 | Extracellular/Cytoplasmic |
| *AdGLP6* | Aradu.B98FL | A02 | 3166 | 1797 | 598 | 7 | 68.33599 | 5.58 | Nuclear |
| *AdGLP7* | Aradu.F1HJA | A02 | 1716 | 1062 | 353 | 2 | 37.58382 | 4.9 | Nuclear |
| *AdGLP8* | Aradu.X3CG0 | A02 | 2728 | 1377 | 458 | 4 | 51.4487 | 5.92 | Chloroplast |
| *AdGLP9* | Aradu.7S7IW | A03 | 3236 | 1314 | 437 | 7 | 48.84029 | 5.82 | Cytoplasmic/Chloroplast |
| *AdGLP10* | Aradu.CFS4Q | A03 | 2369 | 660 | 219 | 2 | 22.89634 | 8.85 | Extracellular/Mitochondrial |
| *AdGLP11* | Aradu.XVQ80 | A03 | 3145 | 1074 | 357 | 3 | 38.48813 | 5.46 | Cytoplasmic/Chloroplast |
| *AdGLP12* | Aradu.3E4UZ | A04 | 847 | 660 | 219 | 2 | 23.2976 | 6.03 | Extracellular |
| *AdGLP13* | Aradu.MA69I | A05 | 1791 | 831 | 276 | 3 | 29.14497 | 7.89 | Chloroplast |
| *AdGLP14* | Aradu.YC8MH | A05 | 2459 | 1266 | 421 | 6 | 47.01672 | 9.04 | Extracellular/Nuclear |
| *AdGLP15* | Aradu.013F5 | A06 | 1260 | 726 | 241 | 2 | 26.17022 | 8.98 | Extracellular/PlasmaMembrane |
| *AdGLP16* | Aradu.0HB4B | A06 | 1203 | 678 | 225 | 2 | 24.24683 | 7.77 | Extracellular |
| *AdGLP17* | Aradu.1QI16 | A06 | 3877 | 1368 | 455 | 4 | 51.21213 | 5.38 | Extracellular |
| *AdGLP18* | Aradu.95XPJ | A06 | 799 | 654 | 217 | 2 | 23.33183 | 6.4 | Extracellular |
| *AdGLP19* | Aradu.F9TAJ | A06 | 8664 | 2631 | 876 | 7 | 100.6231 | 5.64 | Nuclear |
| *AdGLP20* | Aradu.G7AM5 | A06 | 2541 | 1329 | 442 | 4 | 48.78879 | 5.83 | Cytoplasmic/Mitochondrial/Chloroplast |
| *AdGLP21* | Aradu.I953D | A06 | 1897 | 1371 | 456 | 5 | 51.84993 | 6.34 | Cytoplasmic/Nuclear |
| *AdGLP22* | Aradu.K66PA | A06 | 1260 | 741 | 246 | 2 | 26.95323 | 9.35 | Extracellular |
| *AdGLP23* | Aradu.KQ845 | A06 | 2625 | 1536 | 511 | 3 | 54.91243 | 6.6 | Extracellular |
| *AdGLP24* | Aradu.Q9EG1 | A06 | 748 | 675 | 224 | 2 | 24.67652 | 7.1 | Extracellular/PlasmaMembrane |
| *AdGLP25* | Aradu.R35NF | A06 | 1536 | 660 | 219 | 2 | 22.97853 | 9.06 | Mitochondrial |
| *AdGLP26* | Aradu.R9ZWQ | A06 | 651 | 651 | 216 | 1 | 22.62583 | 6.06 | Extracellular/Chloroplast |
| *AdGLP27* | Aradu.W274M | A06 | 956 | 741 | 246 | 2 | 26.88116 | 9.26 | Extracellular |
| *AdGLP28* | Aradu.XDS84 | A06 | 1797 | 1308 | 435 | 4 | 47.88263 | 5.6 | Cytoplasmic |
| *AdGLP29* | Aradu.YBK6Q | A06 | 2518 | 1593 | 530 | 4 | 60.60464 | 5.42 | Nuclear |
| *AdGLP30* | Aradu.YGS80 | A06 | 1876 | 1593 | 530 | 4 | 60.53749 | 5.37 | Nuclear |
| *AdGLP31* | Aradu.B1NGL | A08 | 8145 | 1167 | 388 | 4 | 42.25051 | 5.59 | PlasmaMembrane |
| *AdGLP32* | Aradu.F433U | A08 | 3059 | 1440 | 479 | 5 | 53.89473 | 5.55 | Cytoplasmic |
| *AdGLP33* | Aradu.KB9GU | A08 | 2264 | 1191 | 396 | 3 | 42.8522 | 7.73 | Chloroplast |
| *AdGLP34* | Aradu.VA8CB | A08 | 552 | 552 | 183 | 1 | 19.73536 | 5.16 | Extracellular |
| *AdGLP35* | Aradu.2H0R0 | A09 | 2053 | 1788 | 595 | 5 | 67.80798 | 8.79 | Nuclear |
| *AdGLP36* | Aradu.79I5D | A09 | 4795 | 663 | 220 | 2 | 23.25692 | 7.74 | Chloroplast |
| *AdGLP37* | Aradu.19KPD | A10 | 691 | 618 | 205 | 2 | 22.48747 | 4.99 | PlasmaMembrane |

Note. Chr=Chromosome, MW=molecular weight, pI= theoretical isoelectric point
